# Dibenzoylthiamine Has Powerful Antioxidant and Anti-Inflammatory Properties in Cultured Cells and in Mouse Models of Stress and Neurodegeneration

**DOI:** 10.3390/biomedicines8090361

**Published:** 2020-09-18

**Authors:** Margaux Sambon, Anna Gorlova, Alice Demelenne, Judit Alhama-Riba, Bernard Coumans, Bernard Lakaye, Pierre Wins, Marianne Fillet, Daniel C. Anthony, Tatyana Strekalova, Lucien Bettendorff

**Affiliations:** 1 Laboratory of Neurophysiology, GIGA-Neurosciences, University of Liège, 4000 Liège, Belgium; margaux.sambon@gmail.com (M.S.); juditalhamariba@gmail.com (J.A.-R.); winspierre9@gmail.com (P.W.); 2 Department of Psychiatry and Neuropsychology, Maastricht University, 6200 MD Maastricht, The Netherlands; anna.gorlova204@gmail.com (A.G.); t.strekalova@maastrichtuniversity.nl (T.S.); 3 Institute of Molecular Medicine Laboratory of Psychiatric Neurobiology and Department of Normal Physiology, Sechenov First Moscow State Medical University, 119991 Moscow, Russia; daniel.anthony@pharm.ox.ac.uk; 4 Laboratory for the Analysis of Medicines, CIRM, Department of Pharmacy, University of Liège, 4000 Liège, Belgium; alice.demelenne@uliege.be (A.D.); marianne.fillet@uliege.be (M.F.); 5 Faculty of Sciences, University of Girona, 17004 Girona, Spain; 6 Laboratory of Molecular Regulation of Neurogenesis, GIGA-Stem Cell, University of Liège, 4000 Liège, Belgium; b.coumans@uliege.be (B.C.); b.lakaye@uliege.be (B.L.); 7 Department of Pharmacology, Oxford University, Oxford OX1 3QT, UK

**Keywords:** thiamine, oxidative stress, inflammation, stress, neurodegeneration, animal model, amyotrophic lateral sclerosis, paraquat, lipopolysaccharide, benfotiamine, sulbutiamine

## Abstract

Thiamine precursors, the most studied being benfotiamine (BFT), have protective effects in mouse models of neurodegenerative diseases. BFT decreased oxidative stress and inflammation, two major characteristics of neurodegenerative diseases, in a neuroblastoma cell line (Neuro2a) and an immortalized brain microglial cell line (BV2). Here, we tested the potential antioxidant and anti-inflammatory effects of the hitherto unexplored derivative O,S-dibenzoylthiamine (DBT) in these two cell lines. We show that DBT protects Neuro2a cells against paraquat (PQ) toxicity by counteracting oxidative stress at low concentrations and increases the synthesis of reduced glutathione and NADPH in a Nrf2-independent manner. In BV2 cells activated by lipopolysaccharides (LPS), DBT significantly decreased inflammation by suppressing translocation of NF-κB to the nucleus. Our results also demonstrate the superiority of DBT over thiamine and other thiamine precursors, including BFT, in all of the in vitro models. Finally, we show that the chronic administration of DBT arrested motor dysfunction in FUS transgenic mice, a model of amyotrophic lateral sclerosis, and it reduced depressive-like behavior in a mouse model of ultrasound-induced stress in which it normalized oxidative stress marker levels in the brain. Together, our data suggest that DBT may have therapeutic potential for brain pathology associated with oxidative stress and inflammation by novel, coenzyme-independent mechanisms.

## 1. Introduction

Thiamine (vitamin B1) is an essential micronutrient, indispensable for oxidative energy metabolism, which is especially important for neuronal activity and survival. It is the precursor of thiamine diphosphate (ThDP), an important coenzyme for pyruvate and oxoglutarate dehydrogenases, for transketolase (TKT1) as well as other enzymes [1]. However, other (non-coenzyme) roles of thiamine in the nervous system have been suggested [2,3,4,5].

**Figure 1:**
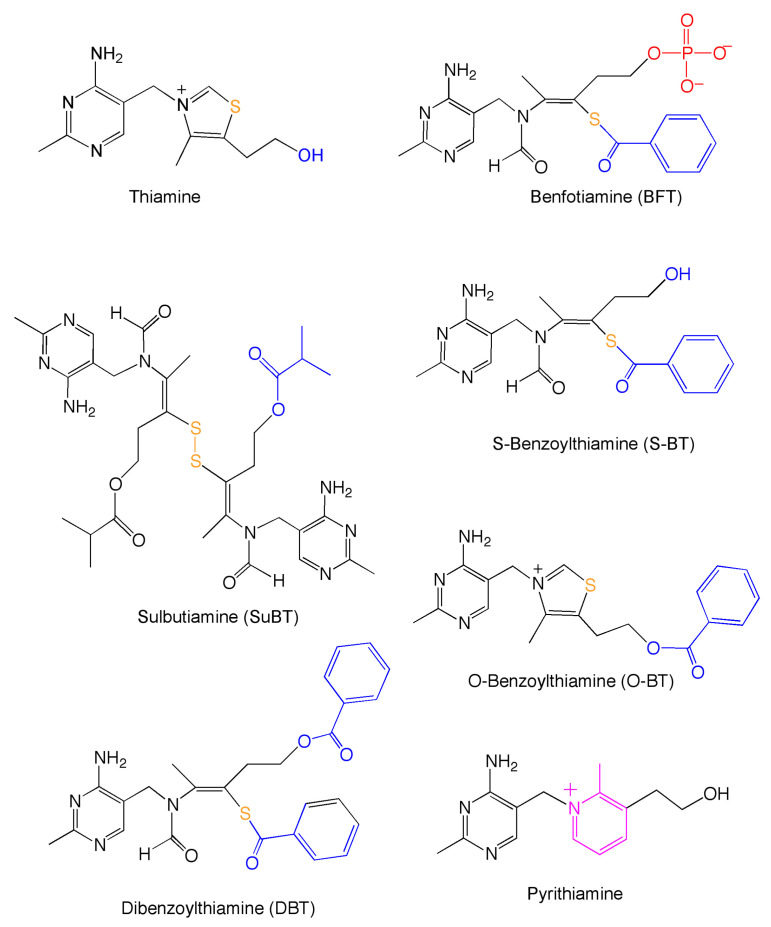
Structural formulas of thiamine, thiamine precursors used in this study, and the thiamine antimetabolite pyrithiamine.

Thiamine deficiency disorders are life-threatening conditions requiring a rapid treatment to restore thiamine levels to physiological values [6]. However, thiamine absorption is limited by the low turnover of high-affinity thiamine transporters; therefore, precursors with a higher bioavailability were developed. These compounds are either hydrophobic or are transformed into hydrophobic thiamine precursors that are able to more rapidly diffuse through the intestinal epithelium into the blood, leading to higher plasma thiamine concentrations. Most of these thiamine precursors were developed in Japan in the 1950s and 1960s and the best known are fursultiamine (thiamine tetrahydrofurfuryl disulfide), sulbutiamine (SuBT), and benfotiamine (BFT) (Figure 1).

More recently, these molecules were shown to have other interesting pharmacological properties. SuBT seems to have antiasthenic properties [7] and it has been claimed that fursultiamine might have some mild beneficial effects in children with a mild autism spectrum disorders [8]. The best studied thiamine precursor is BFT, which, in contrast to SuBT and fursultiamine, is not a disulfide, but a thioester [9]. Moreover, it is not hydrophobic (it is unable to diffuse through cell membranes because of the negatively charged phosphate group) and must be hydrolyzed by intestinal alkaline phosphatase to the hydrophobic S-benzoylthiamine (S-BT, Figure 1). BFT was shown to prevent experimental hyperglycemic damage, probably by reducing the production of advanced glycation end-products (AGEs) [10,11].

Our recent studies have shown that a 2-week pretreatment regimen in mice with thiamine, or BFT, can prevent increases in the GSK3ß activity, overexpression of proinflammatory cytokines and measures of depressive-like or anxiety-like behavior after predator stress or following a swim test [12,13,14]. These compounds also rescued hippocampal neurogenesis and brain protein carbonyl and total glutathione levels after predation stress [3] or after ultrasound-induced stress [15] in mice. The latter study also revealed the superiority of BFT for relieving the negative behaviors. These effects were accompanied by the normalization of the expression of AMPA receptor subunits and plasticity markers [15].

BFT also has powerful beneficial effects in mouse models of neurodegeneration where it has been shown to reduce ß-amyloid deposition, Tau hyperphosphorylation, and improve cognitive performance [16,17]. Lastly, treatment with BFT (300 mg per day for 18 months) has been shown to improve cognitive function in mild to moderate Alzheimer patients that is independent of ß-amyloid accumulation [18].

Given the numerous independent studies that have reported beneficial effects of BFT in human patients and in animal models, it is important to consider what is the mode of action. It was shown that low ThDP levels in blood correlate with brain glucose hypometabolism in Alzheimer patients [19] and it has been suggested that ThDP blood concentrations might be used as a marker for the presence of Alzheimer’s disease [20,21]. Other studies report low plasma thiamine levels in association with mild cognitive impairment in male Parkinson disease patients [22] and beneficial effects of thiamine administration in this disease [23,24]. One cannot exclude the possibility that patients with neurodegenerative diseases may suffer from marginal thiamine deficiency, which would explain some of the improvements observed in previous studies. Indeed, there are some common mechanisms associated with the neurological signs observed in thiamine deficiency and in Alzheimer’s dementia [25]. Nonetheless, it would be a distortion to reduce dementia to a simple thiamine deficiency, and it is worth noting that in animal models of dementia the animals are usually fed on a thiamine-rich diet. Thus, the protective effects described in animals are difficult to interpret and provide little explanation for the mechanisms underlying the neuroprotective effects of BFT.

As the beneficial effects of BFT require treatment with very high doses, 100–200 mg per kg per day for several weeks [16], the therapeutic potential of BFT is limited. Therefore, it would be desirable to find more potent precursors. We investigated the effects of a hitherto unexplored derivative, O,S-dibenzoylthiamine (DBT, Figure 1). DBT has been known for a long time, but there is little information about its properties and biological effects. DBT is allowed as a food additive in Japan, it has a low toxicity, and there is no evidence of carcinogenicity in rats [26]. Moreover, in salmon yearlings, it was shown to be better tolerated than thiamine or BFT; it also led to higher retention of thiamine over time [27].

In this work, we examined the potential protective effects of DBT in vitro and in vivo models. First, we used a simple model consisting of a neuronal cell line and a Nrf2/ARE luciferase reporter 3T3 cell line to study the antioxidant effect of DBT, and a microglial cell line to study its anti-inflammatory effect. In each case, the effects of DBT were compared with those of thiamine, BFT, and SuBT. Second, we studied the effects of the chronic administration of DBT and thiamine in two mouse models: a FUS (Thy-1.FUS 1–359) transgenic mouse model of amyotrophic lateral sclerosis (ALS) and a model of ultrasound-induced stress.

Our data show that DBT is effective in all of the models, in vitro and in vivo, to counteract oxidative stress and inflammation. It was more efficient and acted at lower concentrations than thiamine or BFT, suggesting potential application as a therapeutic in humans. Moreover, we suggest that the biochemical mechanisms underpinning the antioxidant and anti-inflammatory effects are independent of the coenzyme ThDP.

## 2. Experimental Section

### 2.1. Reagents

Thiamine, thiochrome, benfotiamine (BFT), O,S-dibenzoylthiamine (DBT), paraquat dichloride (methyl viologen dichloride hydrate, PQ), lipopolysaccharide (LPS), buthionine sulfoximine (BSO), pyrithiamine hydrobromide (PT), tert-butylhydroquinone (tBHQ), ML385, N-acetyl-L-cysteine (NAC), and benzoic acid (BA) were supplied by Sigma-Aldrich NV/SA (Overijse, Belgium). Sulbutiamine (SuBT) was purchased from Mind Nutrition (https://mindnutition.com). Thiamine-d3 hydrochloride (T344187) and thiamine disulfide hydrate (T344140) were from Toronto Research Chemicals, Toronto, ON, Canada. S-BT and O-benzoylthiamine (O-BT) were gifts from Dr. M. Flint Beal (Brain and Mind Research Institute, Weill Cornell Medicine, New York, NY 10065, USA) [17].

### 2.2. Cells and Cell Culture

Mouse neuroblastoma cells (Neuro2a, RRID:CVCL_0470) and mouse brain microglial cells (BV2 cell line, RRID:CVCL_0182) were first grown in T75 boxes in 10 mL of Dulbecco’s modified Eagle’s medium (DMEM) (Biowest, Riverside, MO, US) supplemented with 10% fetal bovine serum (FBS) (Gibco, Life Technologies Europe BV, Merelbeke, Belgium) in a 5% CO_2_ humidified atmosphere at 37 °C. Before experiments, the cells were grown for 7 days in a custom-made DMEM medium dry-packed without thiamine (Life Technologies Limited, Paisley, Scotland, UK) and 10% FBS. The only source of thiamine was in the FBS and the final concentration of thiamine was found to be around 10 nM, a value close to thiamine concentrations in human plasma. Under these conditions, cells grow normally without signs of thiamine deficiency [28].

The 3T3 cells (Nrf2/ARE luciferase reporter NIH3T3 cell line, Signosis Inc., Gentauer Molecular Products, Kampenhout, Belgium) were stably transfected with pTA-Nrf2-luciferase reporter gene containing four tandem repeats of Nrf2 binding sites. In these cells, the stabilization of Nrf2 leads to its translocation into the nucleus where it binds to the antioxidant response element (ARE) promoter region and initiates the transcription of the luciferase gene. The cells (50,000 cells/well) were grown in 96-well plates for 24 h. After treatment with different concentrations of DBT (25, 50, 100 µM) for 25 h, the cells were washed with 100 µL of PBS and lysed by lysis buffer (Luciferase Cell Culture Lysis, Promega, Leiden, The Netherlands) for 15 min at room temperature. Luciferase substrate (Luciferase Assay System, Promega, Leiden, The Netherlands) was added in each well and the luminescence was measured with a luminometer (GloMax^®^-Multi Detection System, Promega, Leiden, The Netherlands).

### 2.3. HPLC of Thiochrome Derivatives

The method was as previously described [29,30]. For the determination of thiamine derivatives, the cells were grown in 6-well plates. After incubation under various experimental conditions, the cells were washed three times with PBS, scraped in Tris base solution (20 mM, pH 8–8.5), transferred to Eppendorf tubes and sonicated. Cells were treated with trichloroacetic acid (12%) and then centrifuged at 5000× *g* for 15 min. The amount of protein content in the pellet was estimated by the method of Peterson [31] after solubilization in NaOH 0.8 N. The trichloroacetic acid, present in the supernatant, was removed by diethyl ether extraction and the samples were stored at −20 °C.

Prior to analysis by HPLC, samples were oxidized with potassium ferricyanide (4.3 mM, in 15% NaOH) to convert thiamine derivatives to fluorescent thiochrome derivatives. The system was composed of a PRP-1 column (5 μm, 4.1 × 150 mm) protected by a PRP-1 10 μm guard column cartridge (Sigma-Aldrich NV/SA, Overijse, Belgium). The mobile phase was a solution of NaH_2_PO_4_ (50 mM) containing tetra-butylammonium hydrogen sulfate (25 mM) and tetrahydrofuran (4%) at pH 9.5. Thiochrome derivatives were quantified using a fluorescence spectrometer (SFM 25, Kontron Instruments, BRS, Drogenbos, Belgium).

### 2.4. Cell Viability Testing

Cell viability was measured using the (3-(4, 5-dimethylthiazol-2-yl)-2, 5-diphenyl-tetrazolium bromide (MTT) reduction assay (M2003, Sigma-Aldrich NV/SA, Overijse, Belgium). MTT tetrazolium salt was dissolved in serum-free culture medium. The cells were seeded into 96-well plates (50,000 cells per well) and cultured overnight. After incubation under various experimental conditions (for the experimental design see Supplemental Materials and Methods File S1), the cells were incubated in the presence of MTT tetrazolium salt (0.15 mg/mL) for 3 h at 37 °C. Finally, the medium was removed, and dark violet formazan dissolved in a solution of isopropanol and HCl (0.22 N). The absorbance was measured at 580 nm with a Thermo Labsystem plate reader. Results were expressed as percentage of control values.

### 2.5. UHPLC-MS Determination of Metabolites of DBT and Thiamine-d3

Cells were prepared as described in Section 2.2 and diluted four times in H_2_O + 0.1% formic acid. A total of 40 μL of sample was loaded on an Ostro 96-well plate (Waters, Dublin, Ireland) and mixed with 120 μL acetonitrile + 0.1% formic acid to precipitate proteins. Samples were then passed through the plate to remove phospholipids and any residual proteins. Extracts were then vacuum-dried at 30 °C for 90 min and resuspended in 40 µL water + 0.1% formic acid. They were mixed using a CentriVap Concentrator (LabConco, Kansas-City, MO, USA). Calibration curves composed of DBT, SBT, BFT, thiamine, and thiamine d3 were prepared according to the same protocol.

UHPLC was performed on a 1290 Infinity LC system coupled to a 6495 triple quadrupole mass spectrometer (Agilent Technologies, Waldbronn, Germany). Chromatographic separation was performed on a reverse-phase Kinetex F5 column (2.6 µm, 100 × 2.1 mm ID) protected with a Security Guard Ultra F5 precolumn (both from Phenomenex, Torrance, CA, USA) for cells treated with DBT experiments and on a Luna Omega polar C18 (1.6 µm 100 × 2.1 mm) (Phenomenex, Torrance, CA, USA) for cells treated with thiamine-d3 experiments.

The column compartment was thermostated at 40 °C. The separation was carried out in gradient mode with mobile phase A (H_2_O + 0.1% Formic acid) and B (acetonitrile + 0.1% Formic acid) at 0.5 mL/min. The following gradient was used for cells treated with DBT: it started at 2% B and ramped to 40% B in 3 min. It was set at 95% B from 3 to 6 min before being decreased to 2% for 2.5 min. The following gradient was used for cells treated with thiamine-d3 experiments: it started at 0.5% B for 2 min and ramped to 95% B in 3 min. It was set at 95% B from 5 to 7 min before being decreased to 0.5% for 1.5 min. Then, 2 (cells treated with DBT experiments) and 5 µL (cells treated with thiamine-d3 experiments) of sample were injected.

The electrospray source was operated in positive ionization mode (ESI+). The capillary voltage was set at 1500 V. Nitrogen was used as dry gas and sheath gas heated at 210 °C with a flow rate of 17 L/min and 400 °C at 12 L/min, respectively. The nebulizer pressure was settled at 55 psi. The high pressure and low-pressure funnels were operated at 200 and 100 V. Cell accelerator voltage was kept at 4 V. Analyses were conducted in dynamic multiple reaction monitoring (dMRM) mode.

For the experiments with cells treated with DBT, two transitions were followed for DBT and S-BT. The monitored transitions were *m*/*z* 387 → 122 (quantifier) and *m*/*z* 387 → 105 (qualifier) for S-BT and *m*/*z* 491 → 122 (quantifier) and *m*/*z* 491 → 105 (qualifier) for DBT. For the experiments concerning the cells treated with thiamine d3, the monitored transitions were *m*/*z* 268 → 144 for thiamine-d3, *m*/*z* 286 →125 for open thiamine-d3, *m*/*z* 494 → 125 for DBT-d3, *m*/*z* 390 → 125 for SBT-d3.

### 2.6. Protein Carbonyl Content

Carbonylated proteins were detected using the OxiSelect protein fluorometric assay (Cell Biolabs, Bio-Connect Life Sciences, STA-307, TE Huissen, The Netherlands). Neuro2a cells were grown in 6-well plates. The cells were treated with 50 µM of thiamine or DBT for 25 h. After 1 h, PQ (0.25 mM) was added. After treatment, the cells were lysed by addition of the lysis buffer provided in the kit and protein carbonyls in the protein samples were first derivatized with the protein carbonyl fluorophore. Proteins were then precipitated with trichloroacetic acid. The protein pellet was washed with acetone and then dissolved in solubilization buffer. The results were normalized with protein assay (BCA assay 23225, Fisher Scientific, Merelbeke, Belgium). The fluorescence was measured by GloMax^®^-Multi Detection System (Promega, Leiden, The Netherlands).

### 2.7. Thiol Quantification

Free thiols were detected using a protein thiol assay kit (catalog number EIARSHF, Fisher Scientific, Merelbeke, Belgium). This kit detects free thiols in small molecules (reduced glutathione) and protein (cysteine) thiols. Neuro2a cells were grown in 6-well plates. The cells were treated with 50 µM of thiamine or DBT for 25 h. After 1 h, PQ (0.25 mM) was added. After treatment, the cells were lysed by addition of the lysis buffer provided in the kit and then incubated with detection reagent for 30 min, out of direct light. The results were normalized with protein assay (BCA assay). The fluorescence was measured by GloMax^®^-Multi Detection System (Promega, Leiden, The Netherlands).

### 2.8. Reduced Glutathione (GSH) Assay

The levels of reduced glutathione were evaluated using the reduced glutathione (GSH) assay Kit (ab239709, Abcam, Cambridge, UK). Neuro2a cells were grown in 6-well plates. The cells were treated with 50 µM of thiamine or DBT for 25 h. After 1 h, PQ (0.25 mM) was added. After treatment, under various experimental conditions, the cells were lysed by addition of the lysis buffer provided in the kit and centrifuged at 14,000× *g* for 10 min. The GSH in the supernatant reacts with DTNB to generate 2-nitro-5-thiobenzoic acid. This compound is yellow, and the absorbance of sample was measured by GloMax^®^-Multi Detection System (Promega, Leiden, The Netherlands). The results were normalized with protein assay (BCA assay).

### 2.9. NADPH Assay

NADPH was measured using the NADPH assay kit (ab186031, Abcam, Cambridge, UK). Neuro2a cells were grown in 6-well plates. The cells were treated with 50 µM of thiamine, 50 µM DBT, or different concentration of DBT for 25 h. After 1 h, PQ (0.25 mM) was added. After treatment, the cells were lysed by addition of lysis buffer provided in the kit, sonicated and centrifuged at 14,000× *g* at 4 °C for 15 min. The NADPH in the supernatant was detected by adding the NADPH probe. The absorbance of sample was measured by GloMax^®^-Multi Detection System (Promega, Leiden, The Netherlands). The results were normalized with protein assay (BCA assay).

### 2.10. Western Blotting

Neuro2a cells and BV2 cells were grown in 6-well plates. After incubation under various experimental conditions (for the experimental design see Supplemental Materials and Methods File S1), the cells were washed with PBS, scraped in lysis buffer (20 mM Tris-HCl pH 7.5; 150 mM NaCl; 1% Triton X-100; 1 mM EDTA; protease inhibitors (cOmplete, Mini, EDTA-free Protease Inhibitor Cocktail, Roche Diagnostics, Belgium), 1 mM NaF, 1 mM Na_3_VO_4_ and sonicated. They were centrifuged at 16,000× *g* at 4 °C for 15 min and the supernatants were stored at −20 °C. The cell fractionation method used to separate nucleus and cytoplasm was as previously described [32].

Proteins were quantified using BCA assay and equal amounts of protein were electrophoresed through 5–7.5% gels. Proteins were transferred to polyvinylidene fluoride membranes, beforehand activated in 100% methanol, and blocked in 5% nonfat milk TBST (Tris-buffered saline (TBS), 0.1% Tween-20). The membranes were washed three times for 5 min with TBST and exposed overnight to primary antibody at 4 °C. Membranes were then washed three times with TBST and incubated for 1 h with HRP-conjugated secondary antibody. Primary antibody rat anti-glutamate-cysteine ligase catalytic subunit (anti-GCLC, 1:1000, Abcam, Cambridge, UK), primary antibody rat anti-inducible nitric oxide synthase (anti-iNOS, 1:1000, Cell Signaling Technology Europe, B.V., Leiden, The Netherlands), primary antibody rat anti-NF-κB p65 (anti-p65, 1:1000, Cell Signaling Technology Europe, B.V., Leiden, The Netherlands), primary antibody rat anti-nuclear factor (erythroid-derived 2)-like 2 (anti-Nrf2, 1:1000, Cell Signaling Technology Europe, B.V., Leiden, The Netherlands), primary antibody mouse anti-α-tubulin (anti-αtub, 1:5000, Sigma-Aldrich NV/SA Overijse, Belgium), anti-rat and anti-mouse secondary antibodies (1:4000 and 1:10,000, Cell Signaling Technology Europe, B.V., Leiden, The Netherlands and Sigma-Aldrich Sigma-Aldrich NV/SA, Overijse, Belgium) were used. Immunoreactive proteins were detected using a chemiluminescent substrate (kit Western Bright ECL HRP, Advansta—Isogen Life Science, De Meern, The Netherlands) and CDD camera (ImageQuantTM LAS 4000, GE Healthcare Life Sciences, Diegem, Belgium). The signals were quantified with Image J software (version 1.48) and GCLC was normalized with α-tubulin or lamin B.

### 2.11. Quantification of TKT1 Expression by Real Time PCR

Neuro2a cells were grown in Petri dishes (10 cm). The cells were treated with 50 µM of thiamine or DBT for 25 h. After 1 h, PQ (0.25 mM) was added. The mRNA of Neuro2a cells were extracted using the Trizol method and stored immediately at −80 °C. Total RNA (500 ng) was converted into cDNA using random primers and Superscript III transcriptase (Fisher Scientific, Merelbeke, Belgium). Quantitative RT-PCR (qRT-PCR) was performed using the SYBR Green master mix (Roche 4913914001) and lightcycler 480 (Roche). The sequences of the primers for TKT1, housekeeping gene importin 8 (IPO8) and 60 S acidic ribosomal protein P2 (RPLP2) were as reported [33,34]. qPCR measurements were expressed as Cp values (determined by the second derivative method). For each condition, these values were used to determine the relative expression of the gene of interest compared to housekeeping genes using the 2-ΔΔCp method as each gene was amplified with similar efficiency.

### 2.12. Assay of TKT1 Activity

The activity of TKT1 was measured using a kit from Biovision (K2004-100). Neuro2a cells were grown in 6-well plates. The cells were treated with 50 µM of thiamine or DBT for 25 h. After 1 h, PQ (0.25 mM) was added. After treatment, the cells were lysed by addition of lysis buffer provided in the kit and centrifuged at 14,000× *g* at 4 °C for 15 min. Protein concentrations were determined by the BCA assay. Samples were mixed with substrate mix, probe, developer, and enzyme mix. The fluorescence was measured by GloMax^®^-Multi Detection System (Promega, Leiden, The Netherlands).

### 2.13. Nitric Oxide Assay

Nitric oxide was measured using the Griess reagent kit (Promega, Leiden, The Netherlands). BV2 cells were grown in 96-well plates (40,000 cells per well). After incubation under various experimental conditions (described in the Supplemental Materials and Methods), the supernatants of the cells were incubated with sulfanilamide solution for 5 min, then with diazonium salt for 5 min. The absorbance was measured at 560 nm with a Thermo Labsystem plate reader.

### 2.14. TNFα Assay

TNFα produced by BV2 cells was quantified in the culture medium using the TNF alpha Mouse ELISA kit (BMS607-3, Fisher Scientific, Merelbeke, Belgium) according to the manufacturers’ instructions. BV2 cells were grown in 96-well plates (40,000 cells per well). The cells were treated with 50 µM of thiamine or DBT for 25 h. After 1 h, LPS (1 µg/mL) was added. The absorbance was measured at 450 nm with a Thermo Labsystem plate reader.

### 2.15. SLC19A2 (ThTR1) Expression by RT-PCR

Neuro2a cells grown either in normal or thiamine restricted DMEM medium for 7 days were screened for the presence of thiamine transporter 1 (SLC19A2) by RT-PCR. The mRNA was extracted using the RNA isolation Nucleospin^®^RNA XS kit (Macherey-Nagel, Fisher Scientific, Merelbeke, Belgium). Total RNA (1 µg) was converted into cDNA using random primers and Superscript III transcriptase (Invitrogen, Darmstadt, Germany). PCR was performed using a forward primer that spans exons 1 and 2 (5′-GACCGAGAGACAGGTCTACAATG-3′) and a reverse primer that hybridizes at the end of exon 2 (5′-TTCCAC CGGAGGCTCATCTA-3′). PCR products (610 bp) were separated by agarose gel (1%) electrophoresis.

### 2.16. Silencing of Nrf2 Expression by siRNA

The siRNAs directed against Nrf2 were synthesized by the firm Santa Cruz Biotechnology (sc-37049). Under the control conditions, control siRNAs (scrambled sequence) were used (sc-37007, Santa Cruz Biotechnology, TE Huissen, The Netherlands). The siRNAs were diluted in the culture medium at a final concentration of 50 nM and were incubated for 5 min at room temperature. Lipofectamine RNAimax (Fischer Scientific, Merelbeke, Belgium) was used for the transfection of siRNAs. It was diluted in an opti-MEM medium (Fischer Scientific, Merelbeke, Belgium) to a final concentration of 0.002% and incubated for 5 min at room temperature. After this incubation, the siRNAs were mixed with lipofectamine and were incubated for 20 min at room temperature in a 96-well plate. The cells were then seeded at a density of 40,000 in each well. Twenty-four hours after transfection, new siRNAs were added to the cells following the same procedure.

### 2.17. Pharmacokinetics in Mice

Animals were supplied by Charles River Laboratories. Experimental procedures were set up in accordance with the Directive 2010/63/EU and approved by the local veterinarian Committee for Bioethics of Institute of Physiologically Active Compounds of Russian Academy of Sciences (N19-16/06/2017) and Moscow State Medical University (22/10/17-MSMU-35). Male mice, 10–12-week-old, were housed individually under standard laboratory conditions (22 ± 1 °C, 55% humidity, reversed 12 h light/dark cycle with lights on at 19:00, food and water ad libitum). Experimental procedures were set up in accordance with Directive 2010/63/EU. The animals were intraperitoneally injected by a dose of 0, 10, or 50 mg/kg of DBT. The animals were sacrificed after 2, 6, 12, or 24 h. Whole blood, liver, and brain were removed and stored frozen (−70 °C) until use. Thiamine derivatives were determined as previously described [30].

### 2.18. Study with ALS Mouse Model

At the age of 9 weeks, groups of female FUS-tg (Thy-1.FUS 1–359) mice and wild type littermates were treated as described in the Supplemental Materials and Methods File S2. Briefly, administration of DBT (50 mg/kg/day) or thiamine (50 or 200 mg/kg/day), or riluzole (2-amino-6-trifluoromethoxy-benzothiazole, 8 mg/kg/day) was started by a replacement of drinking water with treatment solutions as described elsewhere [12,15,35]. Selective blocker of cyclooxygenase-2 celecoxib (30 mg/kg/day) was administered via food pellets as described elsewhere [35,36]. After 5 weeks of dosing and monitoring of the body weight and liquid intake of the animals, their latency to fall was scored in the wire test on weeks 5 and 6 as described elsewhere [35,37]. The onset of paralysis was recorded in the mutants and the percentage of mice displaying this ALS-like feature at the age of 95 days was calculated for each group.

### 2.19. Study with Ultrasound Model of Emotional Stress

Male Balb/c mice were continuously subjected to unpredictable alternating ultrasound frequencies 16–20 kHz and 20–45 kHz, corresponding to “negative” and “neutral” sounds, which are normally produced by mice, for 3 weeks, as described elsewhere [14,15] (Supplemental Materials and Methods File S3). Concomitantly, subgroups of mice received DBT or thiamine (5, 25, or 200 mg/kg/day) via drinking water, or tap water as described elsewhere [12,14]. Twenty-four hours after the termination of stress exposure, mice were studied in the 6-min swim test and the total duration of floating, a sign of behavioral despair and helplessness, was recorded as described elsewhere [15].

### 2.20. Statistical Analysis

Analysis of the in vitro data was performed using GraphPad Prism software version 5.03 (San Diego California, USA). The results are expressed as mean ± SD or mean ± SEM (in vivo models). Experimental conditions between two groups were made using a Mann–Whitney *U* test. Comparisons between more than two groups were examined by analysis of variance (one-way or two-way ANOVA) followed by appropriate post-hoc tests. Nonlinear correlations were made using Graphpad Prism 8 for MacOS.

In vivo data analysis was performed using GraphPad Prism software version 6.0 (San Diego CA, USA). Data from the study on the FUS-tg mice were examined by two-way ANOVA; data from the experiment with ultrasound stress were analyzed with one-way ANOVA; Tukey’s test was used in the post-hoc analysis. Fisher’s exact test was used to compare the proportion of mice with signs of paralysis at the age of 95 days.

## 3. Results

### 3.1. Treatment with DBT Induces a Rapid Accumulation of Thiamine in Neuro2a Cells Grown in Thiamine-Restricted Medium

The Neuro2a cells were grown in a low-thiamine medium for 7 days before each experiment as previously described [28]. The final concentration of thiamine in this medium supplemented with 10% of fetal bovine serum (FBS) was 10 nM, which is enough for normal cell growth as Neuro2a cells possess high-affinity thiamine transporters [38]. We detected mRNA coding for ThTR-1 in our Neuro2a cells and switching the cells to the DMEM thiamine-restricted medium did not induce a sensible change in THTR-1 expression (Supplemental Figure S1). As this transporter is rapidly saturated, other high capacity, but low-affinity, transporters are responsible for thiamine uptake at concentrations >1 µM [38].

**Figure 2:**
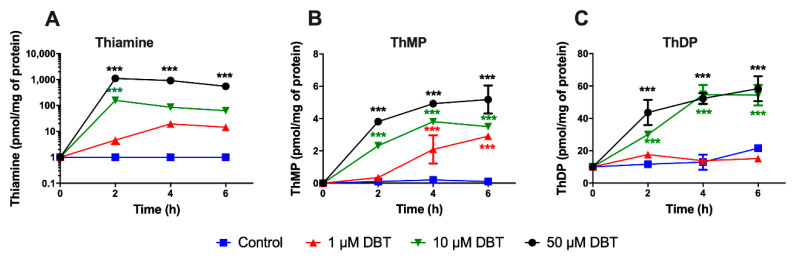
Dibenzoylthiamine (DBT) is a precursor for thiamine and its phosphorylated derivatives in Neuro2a cells. Intracellular contents (pmol/mg of protein) of thiamine (**A**), thiamine monophosphate (ThMP) (**B**), and thiamine diphosphate (ThDP) (**C**) were determined by HPLC as a function of time after exposure to DBT (1, 10, and 50 µM). The controls were made of the usual culture medium without addition of DBT. The data are presented as mean ± SD (*n* = 3). Statistical analysis was made by two-way ANOVA followed by Tukey multiple comparisons test (***, *p* < 0.001).

As the metabolization of DBT has never been reported before, we first studied the transformation of DBT to thiamine, ThMP, and ThDP in Neuro2a cells. The cells were treated with different concentrations of DBT for up to 6 h (Figure 2). The intracellular thiamine, ThMP, and ThDP contents were determined by HPLC. The lipophilic DBT significantly increased the intracellular level of thiamine within 2–4 h and in a dose-dependent manner. The intracellular ThDP content increased more slowly and appeared to reach a maximum of 60 pmol/mg of protein. Indeed, the rate of ThDP synthesis did not increase substantially when the intracellular concentration of free thiamine increased. ThMP content also increased but did not exceed 5 pmol/mg of protein.

On comparing the effects of DBT with those of thiamine, SuBT, and BFT, it appears that DBT, BFT, and SuBT have a comparable ability to increase cell thiamine and ThDP contents. After 25 h, free thiamine is as effective as the three precursors in its ability to raise the ThDP content of the cells (Supplemental Figure S2). It appears that, irrespective of the drug used, ThDP content cannot be raised above a certain level. Hence, the additional effects of DBT (see below) cannot be explained by an ability to increase the cell content of the coenzyme ThDP.

**Figure 3:**
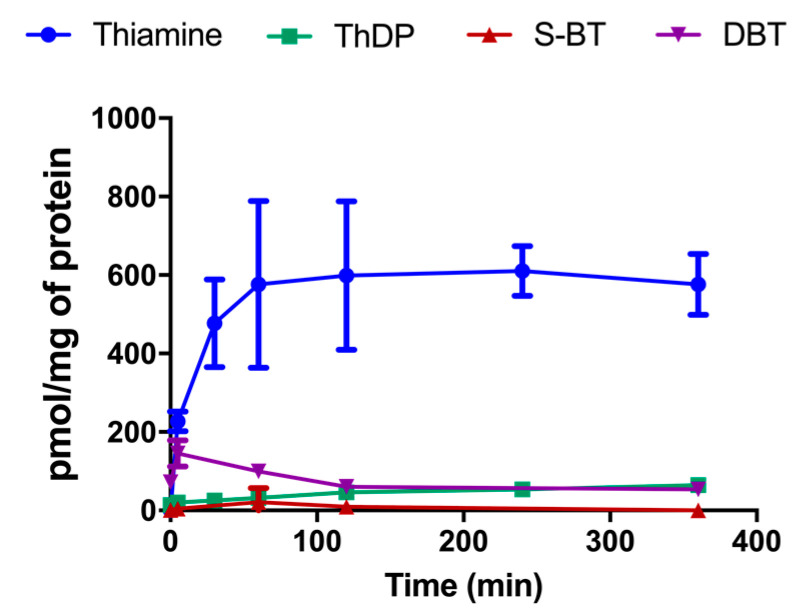
DBT (50 µM) is metabolized to thiamine and ThDP in cultured Neuro2a cells. DBT and S-benzoylthiamine (S-BT) were determined by mass spectrometry and thiamine and ThDP were determined by HPLC as thiochrome derivatives. The data are presented as mean ± SD (*n* = 3).

With our HPLC method, based on the fluorescent detection of thiochrome derivatives, we can only detect compounds that can be directly oxidized to a thiochrome. This is not the case for DBT or the potential metabolites of compounds with an open thiazolium ring. Therefore, we used mass spectrometry to detect such metabolites in the cells after treatment with 50 µM DBT, while thiamine and ThDP were determined by HPLC (Figure 3). In a previous study, we found that the principal metabolite of BFT was S-BT [4]. This did not seem to be the case for DBT, which is much more rapidly transformed to thiamine, without transient accumulation of S-BT. However, only a small fraction (<10%) was pyrophosphorylated to ThDP by thiamine pyrophosphokinase (TPK). This is presumably linked to the fact that TPK is saturated by micromolar concentrations of thiamine (K_m_ = 0.1 µM in Neuro2a cells) and higher amounts do not increase the activity [38].

The deacylation of BFT, DBT, or S-BT by thioesterases requires transit through an open form before yielding thiamine. Using mass spectrometry, we wanted to test whether such an open form could be detected. We did not detect significant amounts of the open form after incubation of the cells with 50 µM BFT or 50 µM DBT. The use of deuterium-labelled derivatives can facilitate interpretation of the data. As deuterium-labelled BFT and DBT were not available, we incubated the cells for 1 h with deuterium-labelled thiamine-d3 (50 µM) and the labelled metabolites were detected by mass spectrometry. Only very minor amounts of the open thiamine-d3, S-BT-d3, and DBT-d3 could be detected (Supplemental Figure S3). This suggests that the open form is very short lived.

It has been suggested that thiamine might be oxidized to thiochrome inside cells. Thiochrome can be detected in samples without derivatization, but we were unable to detect any thiochrome using genuine thiochrome as reference, suggesting that this compound is not formed to any significant amount after treatment with DBT. This is in agreement with previous data using mass spectrometry showing that though small amounts of thiochrome seem to exist in Neuro2a cells, it is not increased after treatment with BFT [4].

**Figure 4:**
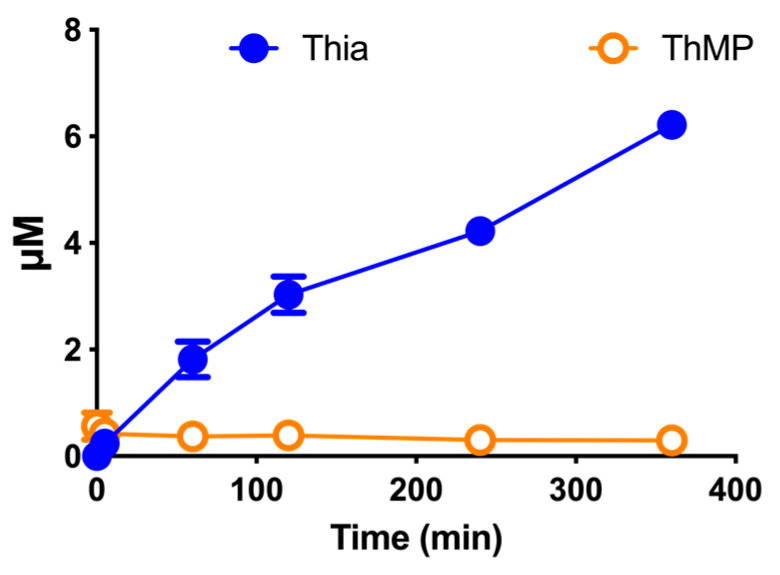
Transformation of DBT (50 µM) in a thiamine-restricted culture medium supplemented with 10% fetal calf serum. The data are presented as mean ± SD (*n* = 3).

To check whether some of the DBT is transformed to thiamine in the culture medium, as previously shown for BFT [4], we incubated the culture medium (without cells) in the presence of DBT (50 µM) for up to 6 h and measured the appearance of thiamine derivatives (Figure 4). After 6 h, the thiamine concentration reached about 6 µM, meaning that about 12% of DBT had been transformed to thiamine. This is much less than for BFT, which had a half-life of about 1 h in the same culture medium [4]. It is thus possible, that after administration of high doses, DBT could transiently accumulate in blood while this is not to be expected for BFT.

### 3.2. Preincubation with DBT Prevents PQ-Induced Toxicity and Oxidative Stress in Neuro2a Cells

**Figure 5:**
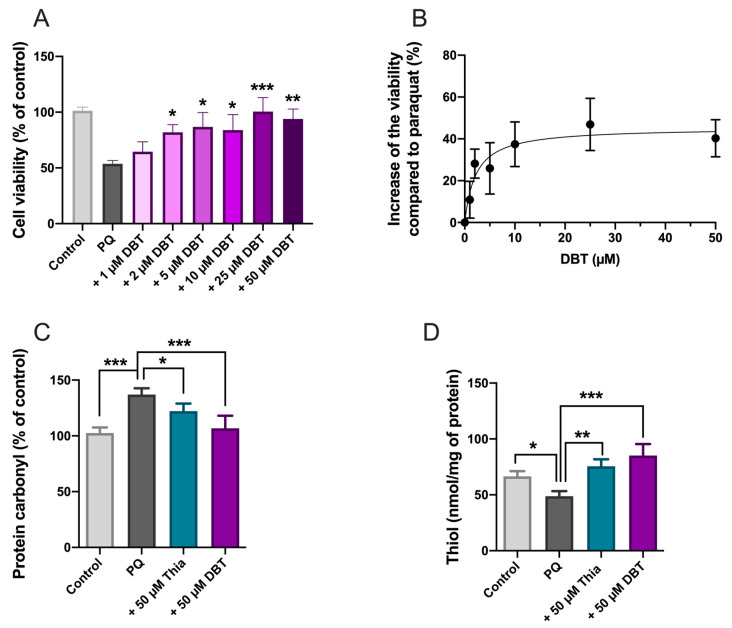
DBT protects Neuro2a cells against paraquat (PQ) toxicity and decreases oxidative stress. (**A**) DBT at various concentrations was added to cells. After 1 h, PQ (0.25 mM) was added (except for the control) and the cell viability (3-(4, 5-dimethylthiazol-2-yl)-2, 5-diphenyl-tetrazolium bromide (MTT) test) was tested 24 h later. (**B**) Dose response curve. The data were fitted using an agonist versus response equation (Y = Bottom + X × (Top-Bottom)/(EC_50_ + X). The results are expressed as mean ± SD (*n* = 3). (**C**) Protein carbonyls (*n* = 4). (**D**) Free thiols (*n* = 3). The results are expressed as mean ± SD. Statistical analyses were made by one-way ANOVA followed by Tukey multiple comparisons test (*, *p* < 0.05; **, *p* < 0.01; ***, *p* < 0.001).

Next, we wanted to test whether DBT can rescue Neuro2a cells from PQ-induced cell death. PQ is a well-known inducer of oxidative stress in various cell types, generating superoxide anions and impairing energy metabolism [39]. In our previous study, we demonstrated that BFT and SuBT (50 and 25 µM) protected Neuro2a cells from PQ-induced cell death [4]. Here we show that DBT is also protective and exerts its effects at lower concentrations. Neuro2a cells were treated with different concentrations of DBT (Figure 5A,B). After 1 h, PQ (0.25 mM) was added and the cell viability was tested 24 h later by means of the MTT test.

Significant protection against PQ toxicity was observed at 2 µM DBT and the EC_50_ was calculated to be 3.4 µM. This finding reveals that DBT is much more effective in this model than thiamine, BFT, or SuBT. Indeed, a significant rescue with BFT or SuBT required a concentration of 25 µM, while thiamine was effective only above 100 µM [4]. In the presence of PQ (0.25 mM) alone, the cell survival rate was approximately 50% that is in agreement with previous results [4]. Note that in the absence of PQ, DBT had no significant effect on cell density (Supplemental Figure S4).

In order to check whether these results can be related to oxidative stress, we measured two indicators of oxidative stress, protein carbonylation (Figure 5C) and cellular content of free thiols (Figure 5D) under the same conditions. PQ increased protein carbonylation, and this carbonylation was antagonized by thiamine and DBT. DBT was also highly efficient in raising free thiol content in Neuro2a cells after PQ treatment. These data clearly show that DBT has antioxidant properties and is more effective than thiamine.

The hydrolysis of the thioester bond of DBT yields O-BT and benzoic acid, which are, therefore, possible metabolites of DBT. Therefore, we tested whether any of these metabolites may have protective effects against PQ (Supplemental Figure S5). While benzoic acid had no significant protective effect, O-BT was relatively efficient, suggesting that O-BT may have either direct protective effects or is the precursor of active metabolites.

### 3.3. Possible Role of Glutathione and NADPH in the Antioxidant Effect of DBT

**Figure 6:**
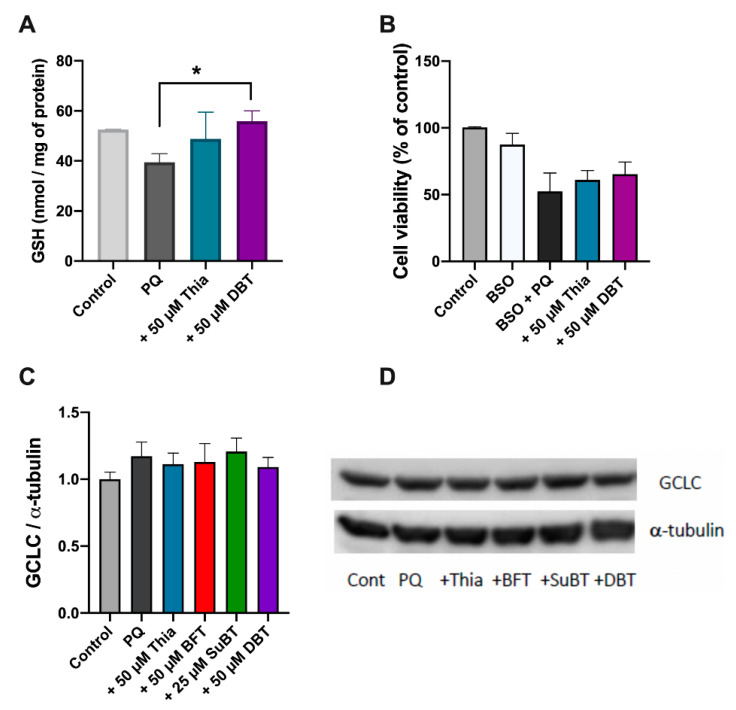
Antioxidant effect of DBT and reduced glutathione (GSH) metabolism. (**A**) Effect of DBT (50 µM, 25 h) on the GSH content in Neuro2a cells treated by PQ (0.25 mM, 24 h). (**B**) Impact of BSO (0.5 mM, 25 h) on the protective effect of DBT (50 µM, 25 h) on PQ-induced cell death (0.25 mM, 24 h). The cell viability was evaluated using the MTT assay. (**C**) Effect of thiamine and thiamine precursors (50 µM, 25 h) on the level of glutamate–cysteine ligase catalytic subunit (GCLC) in Neuro2a cells treated with PQ (0.25 mM, 24 h) using an anti-GCLC antibody. (**D**) An example of an immunoblot. The results are expressed as mean ± SD (*n* = 3 for A, *n* = 4 for B and C). Statistical analyses were made by one-way ANOVA followed by Tukey multiple comparisons test (*, *p* < 0.05).

In most cells, the major intracellular free thiol is reduced glutathione (GSH). Neuro2a cells were treated with 50 µM thiamine or DBT as described above. Figure 6A shows that intracellular GSH levels were reduced by PQ and this reduction was prevented by adding DBT 1 h prior to the addition of PQ. We then tested the effect of BSO, an inhibitor of glutamate cysteine ligase, the enzyme catalyzing the first, and rate-limiting, step of glutathione biosynthesis [40]. BSO significantly reduced the expression of GCLC (Supplemental Figure S6) as well as the cellular levels of GSH, both in the absence and presence of PQ (Supplemental Figure S7). Neuro2a cells were treated with 50 µM thiamine or DBT and 0.5 mM BSO for 1 h. After 1 h, PQ (0.25 mM) was added and cell viability was evaluated after 24 h. In the presence of BSO, no significant rescue of PQ-induced cell death by thiamine or DBT was observed (Figure 6B). We finally tested whether thiamine or its precursors have an effect on GCLC expression using immunoblots, but we were unable to detect any significant effect (Figure 6C,D).

**Figure 7:**
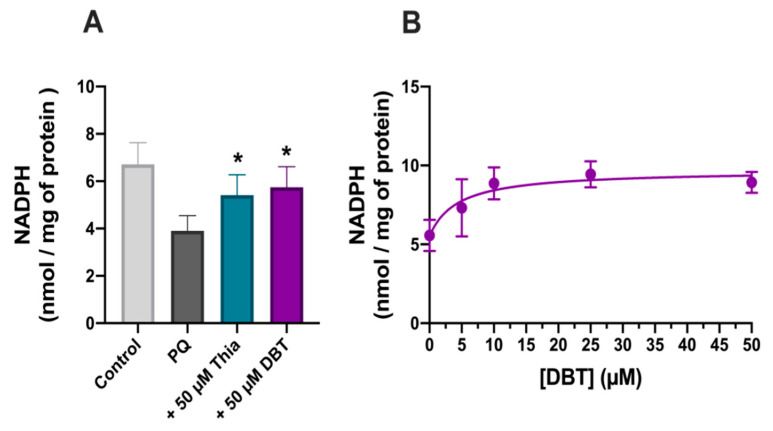
DBT increases NADPH levels in Neuro2a cells treated with PQ (0.25 mM, 24 h). (**A**) Effect of thiamine (Thia) and DBT (50 µM, 25 h) on NADPH level in the presence of PQ. (**B**) Impact of different concentrations of DBT (25 h) on NADPH level in the presence of PQ. The results are expressed as mean ± SD (*n* = 3). The data were fitted using an agonist versus response equation as described in legend to Figure 5 (R^2^ = 0.63). Statistical analyses were made by one-way ANOVA followed by Tukey multiple comparisons test (*, *p* < 0.05).

GSH is regenerated by oxidation of NADPH, mainly derived from the oxidative part of the pentose phosphate shunt. PQ (0.25 mM, 24 h) strongly decreased NADPH levels (Figure 7). This decrease was significantly correlated with cell survival (Supplemental Figure S8). As for thiols and GSH, thiamine and DBT (50 µM, 25 h) antagonized PQ-induced decrease in NADPH levels with an EC_50_ of approximately 4.3 µM (Figure 7B).

**Figure 8:**
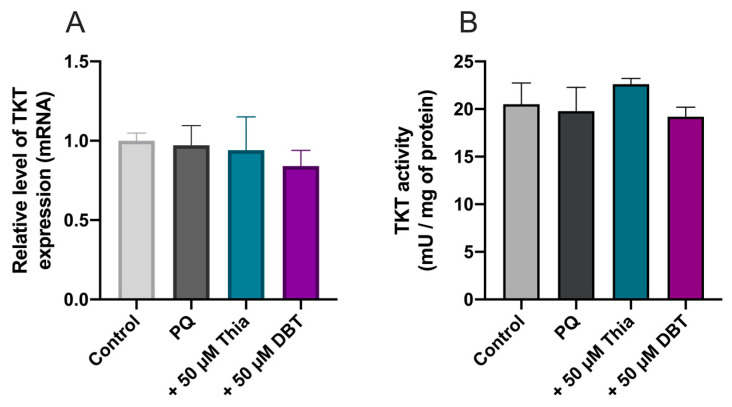
Thiamine (Thia) and DBT (50 µM, 25 h) do not influence the expression (**A**) and the activity (**B**) of transketolase (TKT1) in Neuro2a cells treated with PQ (0.25 mM, 24 h). The relative level of TKT1 mRNA was evaluated by real time PCR. One Unit of TKT1 activity corresponds to 1 µmol of glyceraldehyde 3-phosphate formed per minute at pH 7.5 and at 37 °C. The results are expressed as mean ± SD (*n* = 3). Statistical analysis was made by one-way ANOVA.

Several research papers report increased TKT1 expression or activity induced by BFT (for example see [11,41]). Therefore, we measured TKT1 mRNA levels using real time PCR, but no significant effects were observed (Figure 8A). As TKT1 activity may be increased by Akt phosphorylation [42], we also checked TKT1 enzyme activity, but it was not significantly modified by PQ, thiamine, and DBT (Figure 8B).

### 3.4. Protective Effects of DBT Do Not Involve the Activation of the Nrf2/ARE Pathway

**Figure 9:**
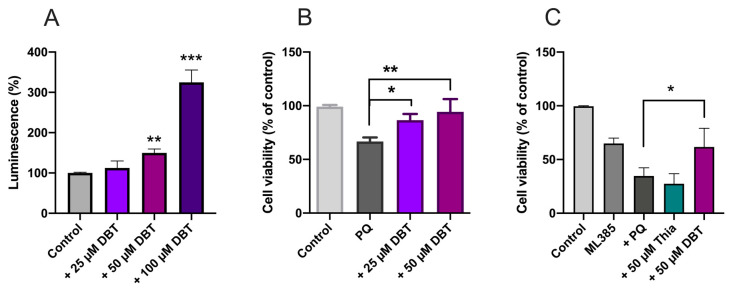
Nrf2/ARE pathway is not implicated in the antioxidant effect of DBT in a Nrf2/ARE luciferase reporter NIH3T3 stable cell line (**A**,**B**) and in Neuro2a cells (**C**). (**A**) 3T3 cells were treated with different concentrations of DBT for 25 h. (**B**) 3T3 cells were treated with 0.25 mM PQ for 24 h in the absence or presence of 25 or 50 µM DBT. (**C**) Impact of ML385 (inhibitor of Nrf2, 10 µM, 25 h) on the protective effect of DBT (50 µM, 25 h) against PQ-induced cell death in Neuro2a cells. The results are expressed as mean ± SD (*n* = 3 for A and B, *n* = 4 for D). Statistical analysis was made by one-way ANOVA followed by Tukey multiple comparisons test (*, *p* < 0.05; **, *p* < 0.01; ***, *p* < 0.001).

Our results clearly show that DBT protects Neuro2a cells against oxidative stress and that this protection might be coupled to NADPH/GSH. Nrf2 is a transcription factor regulating the expression of many genes involved in protection against oxidative stress [43]. Here we used a luciferase reporter stable NIH3T3 cell line to test the effect of DBT on Nrf2 activation. Maximum activation required a DBT concentration of 100 µM, while no significant effect was observed at less than 50 µM (Figure 9A). In contrast, 25 M DBT protected 3T3 cells against PQ-induced cell death (Figure 9B). Hence, much higher concentrations of BFT are required for Nrf2 activation than for protection against PQ-induced cell death, suggesting that the protective effect of low concentrations of DBT involves a different pathway.

In order to confirm this hypothesis, we tested the Nrf2 inhibitor ML385 in Neuro2a cells (Figure 9C). ML385 inhibits the transcriptional activity of Nrf2 by preventing its binding to DNA with an IC_50_ of approximately 2 µM [44]. As this inhibitor was dissolved in DMSO, control experiments were performed at the final DMSO concentration of 0.1% (Supplemental Figure S9). ML385 alone significantly decreased cell survival and addition of PQ decreased the survival by an additional 50%. The effect of PQ was completely prevented by DBT, but thiamine was without effect. As an additional test, we knocked down Nrf2 using a siRNA (Supplemental Figure S10). Though the siRNA strongly decreases NrF2 immunoreactivity DBT was still efficient in preventing the PQ-induced decrease in cell viability. These results further suggest that Nrf2 is not involved in the protection of the cells against PQ-induced cell death by thiamine precursors.

These results also show that the protective effects of DBT are not restricted to Neuro2a cells and are evident in other cell types.

### 3.5. Thiamine and ThDP Have No Direct Antioxidant Effect in Neuro2a Cells

**Figure 10:**
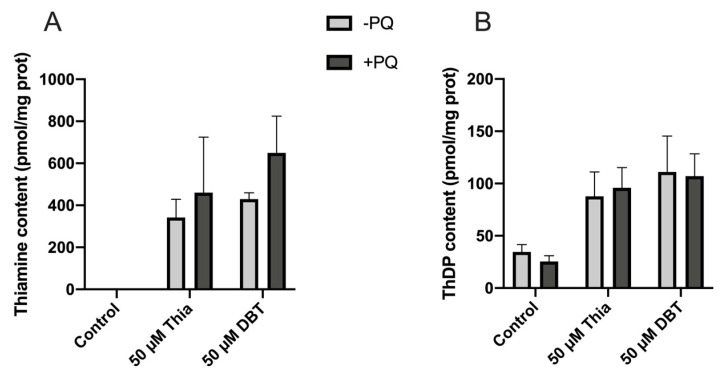
PQ did not affect thiamine (**A**) and ThDP (**B**) content in Neuro2a cells. Neuro2a cells were incubated with or without PQ (0.25 mM) for 24 h at 37 °C in 6-well plates in the absence of added thiamine or in the presence of 50 μM thiamine or DBT. The results are expressed as mean ± SD (*n* = 3). Statistical analysis was made by two-way ANOVA followed by Bonferroni multiple comparisons test. None of the differences between control and PQ were statistically significant.

It has been suggested that thiamine has direct antioxidant properties [45]. This would imply a stoichiometric reaction of thiamine with ROS for instance. Hence, we should observe a disappearance of intracellular thiamine or ThDP during the incubation. However, this was not observed (Figure 10). A reaction between DBT and PQ leading to the neutralization of the latter is also unlikely, as the DBT concentrations are much lower than PQ concentrations. Indeed, cell protection was observed at concentrations less than 10 µM in the presence of 250 µM PQ (Figure 10).

### 3.6. Prophylactic Anti-Inflammatory Effects of DBT in BV2 Cells Activated by LPS

**Figure 11:**
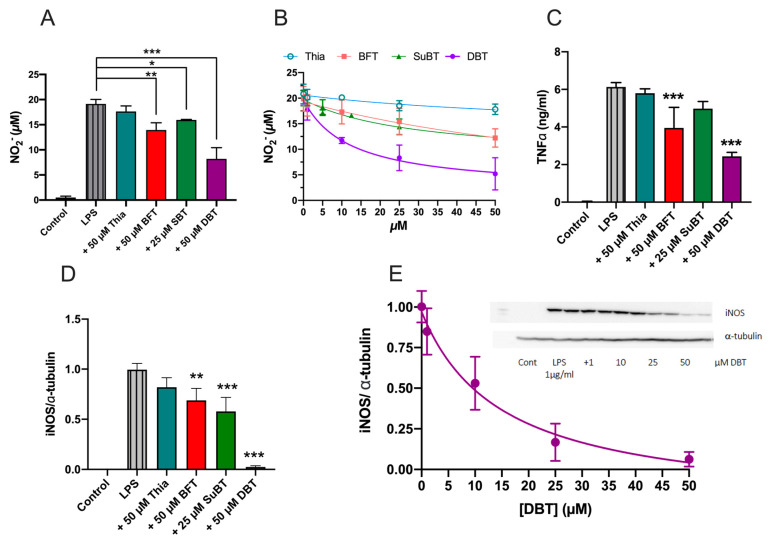
DBT has anti-inflammatory effects in BV2 cells activated by LPS (1 µg/mL, 24 h). (**A**) Effect of thiamine and thiamine precursors (25 h) on NO production in BV2 cells treated with LPS (1 μg/mL, 24 h). (**B**) Effect of different concentrations of thiamine, BFT, SuBT, and DBT on the LPS-induced production of NO in BV2 cells. (**C**) Impact of thiamine, SuBT, BFT, and DBT (25 h) on the LPS-induced production of TNFα in BV2 cells. (**D**) Effect of thiamine, SuBT, BFT, and DBT on the expression of iNOS induced by LPS. The expression of iNOS was evaluated by Western blot and quantified with respect to α-tubulin. (**E**) Dose–response curve for DBT on the expression of iNOS induced by LPS. A nonlinear fit was made using a [Inhibitor] vs. response (three parameters) model. The insert shows a typical immunoblot. The results are expressed as mean ± SD (*n* = 3 for A and B, *n* = 4 for C). Statistical analysis was made by one-way ANOVA followed by Tukey multiple comparisons test (*, *p* < 0.05; **, *p* < 0.01; ***, *p* < 0.001).

As shown for Neuro2a cells and NIH3T3, microglial BV2 cells are also sensitive to PQ poisoning and are rescued by DBT (Supplemental Figure S11). Bacterial lipopolysaccharides (LPS) can induce an inflammatory response in BV2 cells. We studied the anti-inflammatory effects of thiamine and its three precursors BFT, SuBT, and DBT in BV2 cells activated by LPS. BV2 cells were pretreated with thiamine and its precursors for 1 h and then treated with LPS (1 μg/mL) for 24 h. We used three indicators of inflammation: the production of nitric oxide (NO), the level of TNFα, and the expression of inducible nitric oxide synthase (iNOS) (Figure 11). LPS induced an increase in NO_2_^−^ production that was partially antagonized by thiamine precursors, DBT being the most efficient (IC_50_ = 12 µM). Thiamine precursors also antagonized TNFα release and iNOS expression. For all three parameters tested, DBT was by far the most efficient (IC_50_ = 12 µM for NO production and 14 µM for iNOS expression). SuBT significantly decreased iNOS expression but had no significant effect on NO production. Interestingly, while DBT was the most efficient compound in these tests, it was less efficient than BFT in raising intracellular thiamine concentrations (Supplemental Figure S12). This suggests that thiamine is not the compound responsible for the anti-inflammatory properties observed.

O-BT and benzoic acid are possible metabolites of DBT. However, neither compound had any significant effect of NO production in the BV2 cells, suggesting that another metabolite is responsible for the anti-inflammatory effects of DBT (Supplemental Figure S13).

### 3.7. DBT Decreases the Translocation of NF-κB Induced by LPS into the Nucleus of BV2 Cells

**Figure 12:**
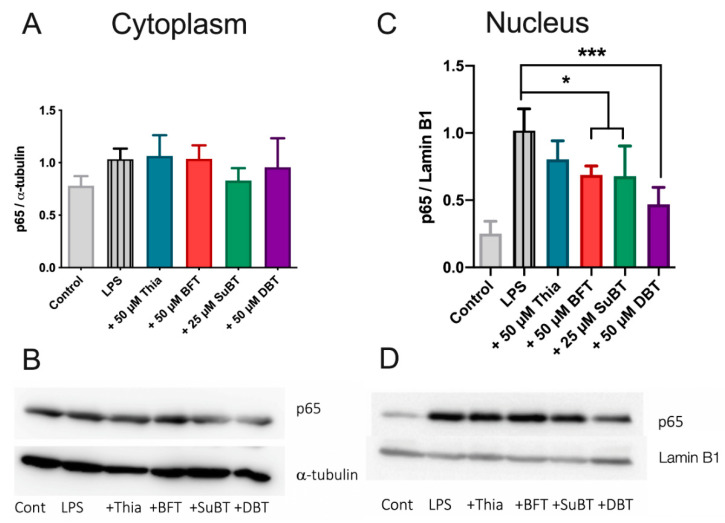
Effect of thiamine precursors on the presence of P65 in the cytoplasmic (**A**,**B**) and nuclear (**C**,**D**) fractions of BV2 cells. The cells were grown in the presence of thiamine precursor for 1 h prior to addition of LPS (1 µg/mL) for 1 h and the level of p65 was evaluated by Western blot. Typical immunoblots are shown in B and D. The signal was normalized with respect to α-tubulin (cytoplasm) and lamin B1 (nucleus). The results are expressed as mean ± SD (*n* = 3). Statistical analysis was made by one-way ANOVA followed by Tukey multiple comparisons test (*, *p* < 0.05; ***, *p* < 0.001).

In BV2 cells, LPS induces the translocation of NF-κB to the nucleus and an increase in proinflammatory genes expression [46]. To determine whether the effects of DBT are mediated by this signaling pathway, we analyzed the influence of thiamine and its precursors on the nuclear translocation of NF-κB (subunit p65) induced by LPS into the nucleus of BV2 cells by Western blotting. BV2 cells were pretreated with thiamine or its precursors for 1 h and then treated with LPS (1 μg/mL) for 1 h. Treatment with thiamine precursors decreases the immunoreactivity of p65 in the nucleus (Figure 12C,D). DBT was the most efficient compound. An antagonization of the translocation of p65 from the cytoplasm to the nucleus by BFT in LPS-treated BV2 cells was suggested in a previous study [47], but in this study the cytoplasmic effects were small. We did not see a significant effect on the level of p65 in the cytoplasm. This is in agreement with another study that showed an LPS-dependent increase of p65 immunoreactivity in the nucleus of BV2 cells without concomitant decrease in the cytoplasm [48]. This may be related to a rapid synthesis of p65 in the cytoplasm.

Overall, our results suggest that DBT alleviates LPS-induced NF-κB activation by preventing translocation of p65 into the nucleus, in agreement with the decreased expression of iNOS and decreased release of TNFα (Figure 11).

### 3.8. Antioxidant and Anti-Inflammatory Effects of DBT Are Not Linked to Increased Cellular Content of the Coenzyme ThDP

**Figure 13:**
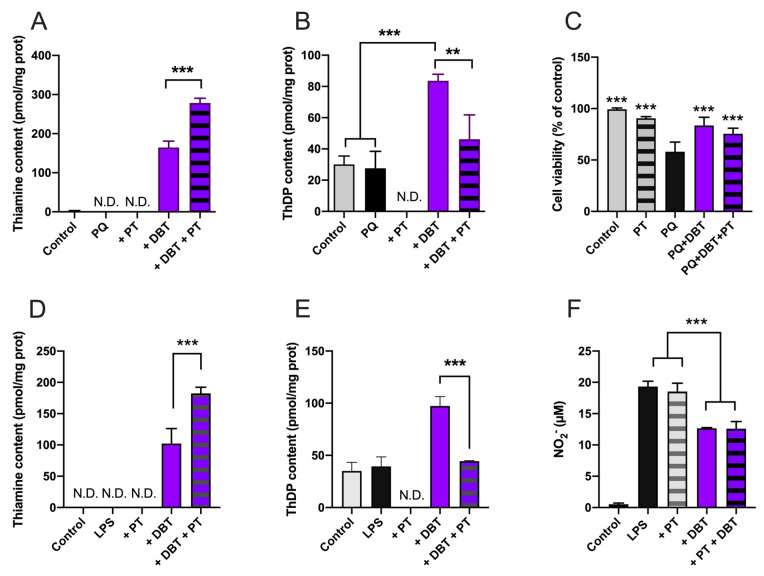
Effect of pyrithiamine (PT) on the intracellular content of thiamine and ThDP and cell survival in the presence of PQ in Neuro2a cells (**A**–**C**) and the intracellular content of thiamine and ThDP or NO production in the presence of LPS in BV2 cells (**D**–**F**). The cells were incubated in the presence of DBT 25 µM with or without PT 25 μM for 25 h. PQ (0.25 mM) or LPS (1 µg/mL) were added 1 h after addition of PT or DBT. The results are expressed as mean ± SD (*n* = 3). Statistical analysis was made by one-way ANOVA followed by Tukey multiple comparisons test (**, *p* < 0.01; ***, *p* < 0.001).

As ThDP is an important coenzyme in cell energy metabolism and for TKT1, we checked whether this compound, generally considered the active thiamine derivative might be involved in the cell-protective effects of thiamine and its precursors. One way to address this question is to use pyrithiamine (PT), a potent inhibitor of thiamine transport [38] and TPK [49], the enzyme responsible for the pyrophosphorylation of thiamine to ThDP. In the presence of DBT, which does not require a transporter to enter the cells, the only effect of PT is the suppression of ThDP synthesis by inhibition of TPK. Treatment of the cells with PT (25 µM, 25 h) in the presence of DBT led to increased intracellular thiamine (Figure 13A,D) and decreases of ThDP to control levels (Figure 13B,E) in Neuro2a and BV2 cells. However, DBT was still able to increase cell survival in Neuro2a cells (Figure 13C) and still decreased NO production in BV2 cells (Figure 13F) to the same extent as in the absence of PT. We previously obtained similar results with BFT in place of DBT in Neuro2a cells [4]. These results strongly suggest that the coenzyme ThDP is not involved in cell-protective mechanisms after PQ or LPS treatment.

### 3.9. Effect of DBT Administration on the Content of Thiamine Derivatives in Mice

**Figure 14:**
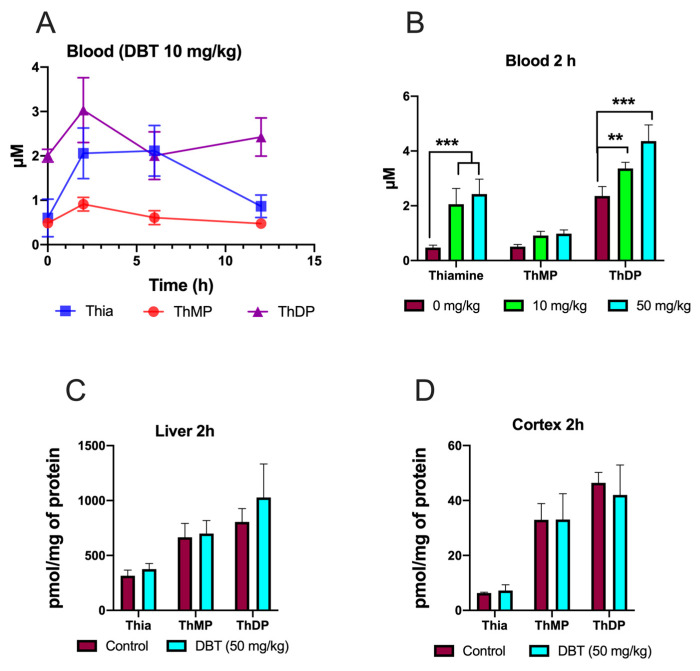
Effect of DBT administration on the content of thiamine derivatives in mice. (**A**) The mice were injected with a dose of 10 mg/kg and thiamine derivatives were determined in whole blood after 2, 6, and 12 h. (**B**) The mice received a dose of either 0, 10, or 50 mg/kg and thiamine derivatives were determined in blood after 2 h. (**C**) The mice received a dose of 50 mg/kg and thiamine derivatives were determined in liver after 2 h. (**D**) The mice received a dose of 50 mg/kg and thiamine derivatives were determined in brain cortex after 2 h. The results are expressed as mean ± SD (*n* = 3). Statistical analysis was made by two-way ANOVA followed by Tukey (A–C) multiple comparisons test (**, *p* < 0.01; ***, *p* < 0.001).

Mice were given a dose of DBT (10 mg/kg) (intraperitoneal injection) and thiamine derivatives were determined in blood after 2, 6, or 12 h (Figure 14A,B). The maximum levels were obtained after 2 h, which is in agreement with previous data obtained with BFT [9]. The main difference for BFT is that high ThDP (which is mainly localized in red blood cells) levels were maintained for a longer time with DBT compared to BFT. In agreement with the data obtained with BFT [3,9,16,17], no significant increase in ThDP content was observed in liver and brain tissues (Figure 14C,D).

### 3.10. Administration of DBT but Not Thiamine Ameliorates ALS-Related Motor Dysfunctions

**Figure 15:**
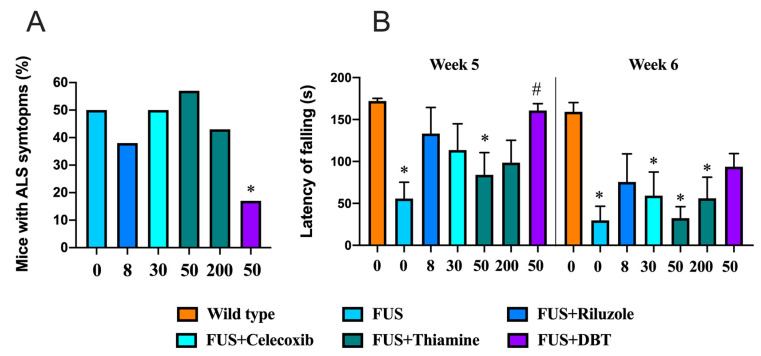
The administration of DBT ameliorates motor dysfunction in FUS-tg mice. (**A**) In 95-day old mice, fewer FUS-tg mutants displayed ALS-like paralysis in the group that received DBT, but not in the mice that received thiamine (*, *p* < 0.05 vs. vehicle-treated FUS-tg mice, Fisher’s exact test). (**B**) On week 5 of the experiment, FUS-tg mice treated with DBT displayed a longer latency to fall in the wire test than the vehicle-treated mutants. Mutants that received vehicle or thiamine at the dose 50 mg/kg/day exhibited a decreased latency to fall in comparison with wild type mice. On week 6, all FUS-tg groups except the riluzole- and DBT-treated mutants, displayed a significant reduction in the latency to fall in comparison with wild type mice (*, *p* < 0.05 vs. wild type mice, #, *p* < 0.05 vs. vehicle-treated FUS-tg mice. Two-way ANOVA, Tukey’s post hoc test). *x*-axis: doses are expressed in mg/kg/day. DBT—dibenzoyl thiamine. A total of 7–16 animals per group were used. Bars: mean ± SEM.

In the next step we sought to examine the effects of DBT in animal models. Employing the FUS-tg mice, a genetic model of ALS, we found that the percentage of FUS-tg mice with paralysis recorded at 95 days was significantly lower in the group of mice treated with DBT than in vehicle-treated FUS-tg mice (*p* = 0.0434, Fisher’s exact test; Figure 15A). No significant difference in the level of paralysis was found in mutants treated with riluzole, celecoxib, or the lower or higher doses of thiamine compared to the vehicle-treated FUS-tg control group (*p* = 0.167, *p* > 0.999, *p* > 0.999, and *p* > 0.999, respectively; Fisher’s exact test). Riluzole was chosen as a pharmacological reference for use in this study, as it is currently a standard-of-care treatment for ALS that has been shown to arrest disease progression for 2–3 months [35].

A two-way ANOVA revealed significant effects of genotype, treatment, and their interaction on the latency to fall in the wire test on week 5 (F = 21.6, *p* < 0.0001; F = 8.23, *p* = 0.0372; F = 9.87, *p* = 0.0155, respectively). The vehicle-treated FUS-tg mice and FUS-tg mice that received thiamine at the dose of 50 mg/kg/day demonstrated a significant reduction the time to fall in comparison to wild type (*p* < 0.0001 and *p* = 0.0012, respectively, Tukey’s test; Figure 15B). However, the FUS-tg mice treated with DBT exhibited a significant increase in the latency to fall in comparison with the vehicle-treated FUS-tg mice (*p* < 0.0001, Tukey’s test); no other group differences were found. On week 6, in comparison with wild type group, the latency to fall was significantly decreased in the vehicle-treated FUS-tg mice, and the FUS-tg mice given celecoxib or thiamine at the doses of 50 and 200 mg/kg/day (*p* = 0.0061, *p* = 0.0001, and *p* = 0.004, respectively). No such difference was found in FUS-tg mice treated with riluzole or DBT (*p* = 0.072 and *p* = 0.065, respectively). Thus, treatment with DBT, but not thiamine, arrested the development of ALS-like motor deficits in the FUS-tg mice.

In wild-type mice, treatment with DBT, thiamine, riluzole, and celecoxib did not affect the latency to fall in the wire test on weeks 5 and 6 of the study (Supplemental Figure S14).

### 3.11. Greater Normalizing Effects of DBT than of Thiamine on Depressive-Like Behavior in the Ultrasound-Induced Stress Model

**Figure 16:**
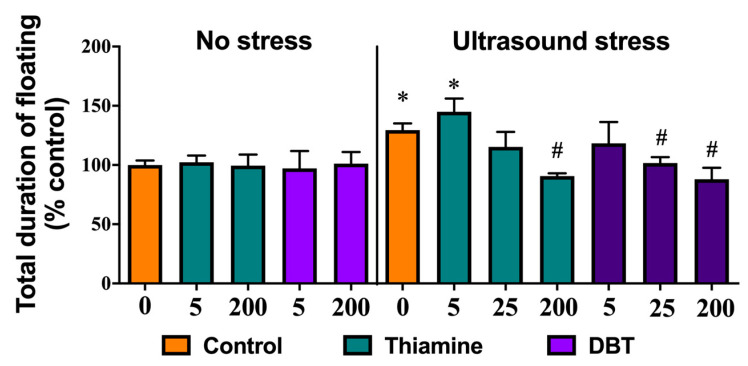
The administration of low doses of DBT, but not thiamine prevents the stress-induced increase of helpless behavior in mice. In the forced swim test, in comparison to control group, ultrasound-exposed vehicle-treated mice and stressed mice treated with thiamine at the dose of 5 mg/kg/day showed a significant increase of the total duration of floating. Ultrasound-exposed groups treated with thiamine at the dose of 200 mg/kg/day and DBT at the dose of 25 or 200 mg/kg/day displayed significantly shorter duration of this behavior than vehicle-treated mice exposed to the ultrasound stress (*, *p* < 0.05 vs. nontreated control mice, #, *p* < 0.05 vs. vehicle-treated ultrasound-exposed group, one-way ANOVA and post hoc Tukey’s test). A total of 6–10 animals per group were used. Doses are expressed in mg/kg per day. Bars are mean ± SEM.

In the second set of in vivo experiments with DBT, an ultrasound exposure was used to generate depressive-like behavior that was evaluated by measuring floating behavior in a swim test. One-way ANOVA revealed a significant difference between groups in the total duration of floating between the stressed and unstressed mice (F = 3.728, *p* = 0.0002). Post-hoc analysis showed that there was a significant increase in the duration of floating in the ultrasound-exposed vehicle-treated group and in the ultrasound-exposed group that was treated with the lowest dose of thiamine, as compared to control groups (*p* = 0.0218 and *p* = 0.0074, respectively, Tukey’s test; Figure 16). In comparison with vehicle-treated stressed mice, the total duration of floating was significantly decreased in stressed animals treated with thiamine or DBT at the dose of 200 mg/kg/day (*p* = 0.036 and *p* = 0.0172, respectively), and in mice that received DBT at the dose 25 mg/kg/day (*p* = 0.039), but not in stressed mice that received thiamine at that dose (*p* = 0.975). Lower doses of thiamine or DBT (5 mg/kg per day) did not significantly reduce the duration of floating (*p* = 0.998). Thus, the administration of DBT at 25 mg/kg per day was effective in preventing stress-induced induction of helpless behavior in mice, while the administration of equivalent doses of thiamine was ineffective.

Thus, ultrasound stress induced floating behavior, a sign of ‘despair’ and helplessness, while treatment with both thiamine and DBT improved this outcome by reducing this measure. However, thiamine was effective only at the dose 200 mg/kg/day, while a lower dose of 25 mg/kg per day of DBT significantly reduced duration of floating.

As it is known that ultrasound stress generates oxidative stress in mouse brains, and, as this stress is reduced by administration of thiamine and BFT [15], we tested whether DBT decreases oxidative stress in this model using malonaldehyde as the stress marker. Our results show that malonaldehyde content is indeed increased in the hippocampus of the animals subjected to ultrasound stress and that it was decreased by DBT, which seemed to be more effective than thiamine (Supplemental Figure S15).

## 4. Discussion

Oxidative stress and neuro-inflammation are prominent features of neurodegenerative diseases and possibly of other disorders such as major depression.

Several studies showed that BFT, a synthetic precursor of thiamine, has therapeutic value in mouse models of neurodegeneration (Alzheimer, tauopathies) [16,17] but also in mouse models of depression related to chronic stress [3,15]. In these models, BFT reduced oxidative stress and inflammation, but its mechanism of action is unclear. As the animals are fed a thiamine-rich diet, it is unlikely that the effects are attributable to the remediation of a thiamine deficiency. Moreover, the treatment with BFT does not induce an increase in brain levels of the coenzyme ThDP and ThDP-dependent enzyme activities are only slightly, if at all, increased. Therefore, BFT does not seem to boost oxidative metabolism, and more specific pharmacological effects must be responsible for its mode of action.

In rodents, treatment with BFT requires relatively high doses, optimally in the range of 100–200 mg per kg per day, for several weeks, which is much more than is required amount to relieve thiamine deficiency symptoms [16,17]. It is therefore desirable to find compounds that can act at lower doses. In this respect, DBT is a promising candidate as, in contrast to BFT (which is not lipophilic and must be dephosphorylated by intestinal phosphatases), it is lipophilic and crosses biological membranes by simple diffusion as we have confirmed here (Figure 3). Moreover, although no pharmacological study is available, DBT has been shown to be nontoxic and nontumorigenic [26] and it is permitted as a food additive in Japan.

Here, we show for the first time that DBT is protective in Fus-tg mice, a mouse model of ALS, and in a mouse model of stress in which DBT exhibited an antidepressant action, possibly by reducing oxidative stress. Data from the FUS-tg mice demonstrate that chronic administration of DBT, but not thiamine (used at four-fold higher dose than DBT), increases the time to the onset of paralysis, a hallmark of pathology in this model [35,50]. Moreover, the effects of DBT were more pronounced than those of the standard ALS treatment riluzole. In keeping with previous studies, effects of celecoxib administration were limited [35]. Our experiments with an ultrasound-induced stress model showed that both thiamine and DBT decreased the duration of floating in the forced swim test, a sign of decreased behavioral despair in stressed mice. Moreover, the administration of a low dose (25 mg/kg per day) of DBT was effective in decreasing depressive-like behaviors in stressed mice, whereas only the dosing with high concentration of thiamine (200 mg/kg per day) is able to prevent these changes. Together, these data demonstrate an increase in biological potency with DBT in comparison to thiamine in the two mouse models that both exhibit increases in oxidative stress markers in the brain. They also suggest that, as hypothesized above, DBT might act at lower concentrations than BFT. This raises the question of what the molecular mechanisms are and what are the active metabolite(s) involved in the DBT-induced improvement in outcomes.

To explore the mode of action of DBT, we used simple cellular models. Considering that it has already been demonstrated that BFT has antioxidant effects in Neuro2a cells [4] and anti-inflammatory effects in LPS-stimulated BV2 cells [47], we tested DBT in the same cellular models. In each case, the effect of DBT was investigated with comparison to thiamine, BFT, and SuBT, which is in contrast to most other studies that test only one compound. In addition, we sought to identify other possible mechanisms of action. Our results demonstrate that pretreatment with DBT decreases oxidative stress in Neuro2a cells treated by PQ and inflammation (production of NO, TNFα, expression of iNOS) in BV2 cells activated by LPS. DBT was generally more effective and acted at lower concentrations than BFT, but its mechanism of action seems to be the same. It is well known that the Parkinsonian mimetic PQ induces the production of ROS, but its mechanism of action is complex and may result from ROS-induced impairment of mitochondrial permeability [51]. The toxic effects of PQ have been counteracted by inhibitors of NADPH oxidase (NOX). Interestingly, it has been shown that BFT reduces NOX4 expression in human myotubes, which would result in increased NADPH levels [52]. NOX4 also stimulates Nrf2 translocation into the nucleus, thereby boosting expression of GSH synthesizing enzymes. However high concentrations of BFT (100–200 µM) were required to downregulate NOX4 expression, which is not the case in our model, where 25 µM BFT were protective [4]. This suggests that a different mechanism is involved. We also showed that Nrf2 is not involved in the protective effects generated by low concentrations of DBT.

Here, we showed that the antioxidant effect of DBT in Neuro2a cells seems to be dependent on GSH, the most abundant antioxidant molecule. Indeed, an inhibition of GSH synthesis counteracts the protective effect of DBT (Figure 6). NADPH, which regenerates reduced GSH, seems to play a role, suggesting the involvement of the oxidative part of the pentose phosphate shunt. Some studies have shown increased TKT1 expression and enzymatic activity and suggest that this could induce increased flux through the oxidative part of the shunt leading to increased production of NADPH [44]. In our work, PQ did not decrease TKT1 expression and enzyme activity and, at 50 µM, thiamine and DBT had no significant effect on TKT1 expression and enzyme activity in Neuro2a cells (Figure 8). This result is important, as TKT1 expression is tightly regulated by Nrf2 [53], a master regulator of many genes involved in cell protection against oxidative stress. Tapias et al. [17] showed that the activity of the Nrf2/ARE pathway is increased by high concentration of BFT (100–200 µM), in agreement with our data (Figure 9). However, DBT had protective effects at low concentration (<25 µM) not inducing activation of this pathway as well as in the presence of ML385, inhibiting translocation of Nrf2 into the nucleus.

It has also been shown that Akt may activate TKT1 by phosphorylation, thereby increasing the flux through the nonoxidative branch of the shunt [42]. Our results show that neither thiamine nor DBT increased TKT1 enzyme activity at 50 µM (Figure 8).

In our study, the commercial kit, used for measuring TKT1 activity, already contained ThDP. Hence, it was not possible to estimate the degree of saturation of the enzyme by its coenzyme. However, as the oxidative and nonoxidative branches of the shunt are considered to be relatively independent, and changes in TKT1 activity are not thought to markedly affect the oxidative branch. Indeed, in a recent study, silencing TKT1 expression by 80% led to a 50% decrease in TKT1 activity, but hardly affected the oxidative branch in a human breast cancer cell line [54]. As already suggested for BFT, the coenzyme ThDP does not seem to be involved: we showed that in the presence of pyrithiamine, no net ThDP synthesis occurred during incubation with DBT, but its protective effects were maintained both in Neuro2a and BV2 cells (Figure 13). It was recently suggested that ThDP could have an antioxidant effect that is independent of its coenzyme role [55]. DBT was no more efficient in raising intracellular ThDP concentrations than thiamine, SuBT, or BFT in Neuro2a (Supplemental Figure S2) and BV2 cells (Supplemental Figure S12), though it was pharmacologically more active. Therefore, it is doubtful that the effects observed here are mediated by ThDP.

Hence, with respect to the antioxidant effects, low concentrations of DBT seem to activate the oxidative part of the pentose phosphate shunt, increasing the production of NADPH and GSH.

**Figure 17:**
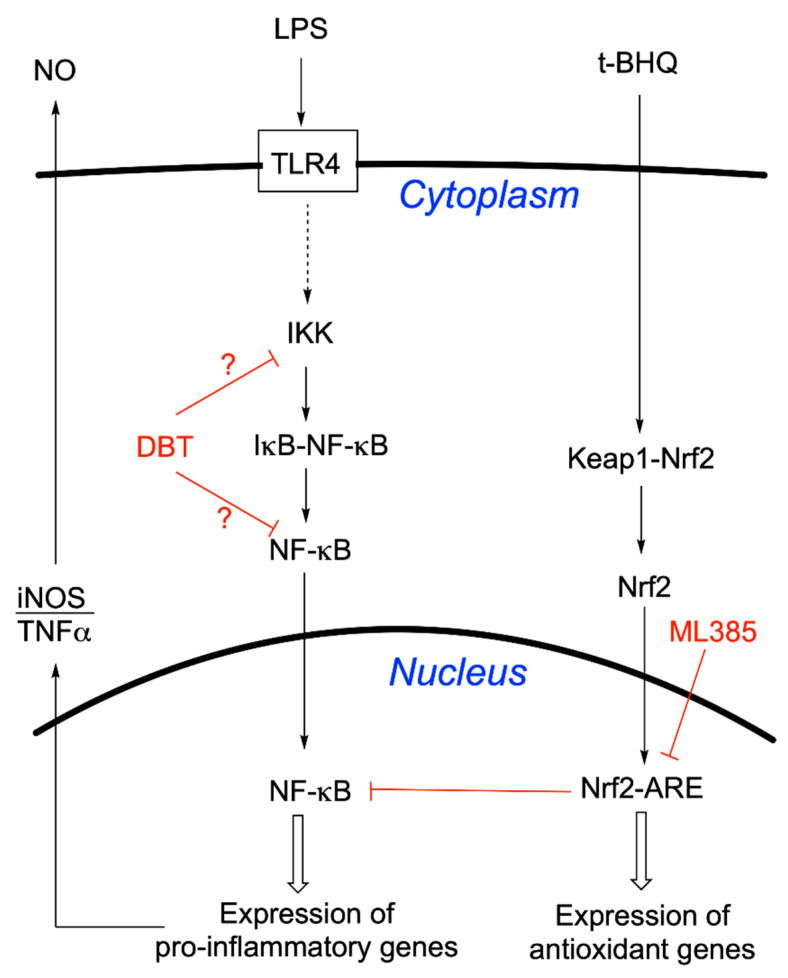
Possible mechanism for the anti-inflammatory effect of DBT in microglial BV2 cells. According to this model, an active metabolite of DBT blocks (red lines) the activation of the transcription factor NF-κB either by inhibiting the phosphorylation of IκB (which induces the release of NF-κB) or by acting directly on the complex IκB-NF-κB preventing its dissociation. ML385 inhibits (red lines) Nrf2 transcriptional activity which in turn results in decreased NF-κB expression. TLR4—Toll-Like Receptor 4; IKK—IκB kinase; t-BHQ—tert-butylhydroquinone, a specific activator of the Nrf2/ARE pathway.

Concerning the anti-inflammatory effects in BV2 cells, LPS is known to activate NF-κB signaling, by its translocation into the nucleus and an increase of proinflammatory genes expression. Our results show that DBT inhibits the translocation of NF-κB into the nucleus and so counteracts the increase of transcription of proinflammatory genes (Figure 17). This is in agreement with published data reporting that BFT (50–100 µM) prevented NF-κB activation in murine macrophages [56], but our results show that DBT is more efficient and active at lower concentrations (Figure 11 and Figure 12). This may be of importance as neuroinflammation is a major contributor to neurodegeneration. The link between neuroinflammation and neurodegenerative diseases is underlined by recent findings that genetic variants of immune receptor genes, including TREM2, a gene expressed only by microglia among CNS cells, are associated with Alzheimer’s disease, frontotemporal lobar degeneration, and possibly Parkinson’s disease [57]. Misfolded and aggregated proteins in neurodegenerative diseases bind to pattern recognition receptors on microglia and astroglia cells and lead to their activation. Hence, excessive activation of glial cells by inflammation contributes to disease progression and severity.

Our results suggest that the mechanisms of DBT action are different for antioxidant and anti-inflammatory effects, the former involving GSH and NADPH, the latter NF-κB activation. A further argument in favor of different mechanisms comes from the observation that N-acetylcysteine, a precursor of cysteine which itself is a substrate of GCL, catalyzing the rate limiting step in the synthesis of GSH, protects Neuro2a against PQ-induced oxidative stress, but not against NO production, a marker of inflammation (Supplemental Figure S16). However, much higher concentrations of N-acetylcysteine than of DBT are required.

**Figure 18:**
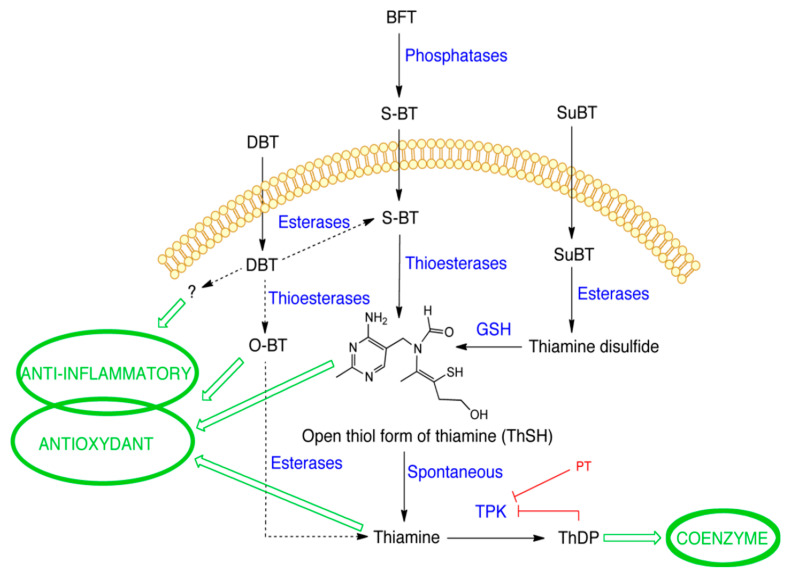
Metabolism of thiamine and its precursors (DBT—dibenzoylthiamine; BFT—benfotiamine; S-BT—S-benzoylthiamine; SuBT—sulbutiamine; ThTR—thiamine transporter; PT—pyrithiamine; O-BT—O-benzoylthiamine; ThSH—thiol (open) form of thiamine; ThDS—thiamine disulfide; GSH—reduced glutathione; TPK—thiamine pyrophosphokinase; ThDP—thiamine diphosphate).

This raises the question as to the nature of the active compound(s) involved in the DBT protective effects, a point that is intimately related to the metabolization of this precursor molecule (Figure 18). As we could exclude direct antioxidant effects of thiamine and ThDP (Figure 10), we considered the possibility that the cell-protective effects of DBT are linked to its ability to increase the intracellular levels of the coenzyme ThDP (Figure 2C). However, thiamine was just as effective as DBT in raising intracellular ThDP concentrations both in Neuro2a and BV2 cells (Supplemental Figures S2 and S12), while it was much less protective (Supplemental Figure S11) against PQ and it had no anti-inflammatory properties (Figures S11 and S12). Moreover, the protective properties of DBT were maintained when ThDP synthesis was blocked by pyrithiamine (Figure 13). This is also in agreement with in vivo data in mice showing beneficial effects of BFT without increase in brain ThDP content [3,16,17]. These results suggest that the protective effects of DBT and BFT are not linked to the coenzyme ThDP.

We have previously shown that the metabolization of BFT proceeds via formation of S-BT, which is readily transformed into thiamine [4]. This is in agreement with data obtained by others, showing that the metabolization of S-BT mainly yields thiamine and a variable proportion of O-BT [58]. This transformation seems to be the driving force for transmembrane diffusion of S-BT into the cells. Enzymatic deacylation of S-BT by thioesterases might be predominant in the intracellular medium.

With DBT, we did not observe a strong accumulation of S-BT, one of the principal degradation products of BFT [4]. It is possible that the hydrolysis of DBT to S-BT is much slower than the subsequent hydrolysis of S-BT and formation of thiamine. Indeed, our results suggest that DBT is more stable than BFT in the DMEM medium with 10% FBS (Figure 4), compared to BFT [4]. This could also mean that it is possible that in in vivo models, significant amounts of DBT may be carried by the blood stream and could, ultimately, enter the brain.

In our cultured cells (and also in brain cells), we cannot exclude the presence of other degradation products. The nonthiamine moiety of DBT, benzoic acid is almost quantitatively excreted by the kidneys as hippuric acid after oral administration of DBT in rats [59] and it did not demonstrate protective effects in this study. Another possibility would be O-BT (Supplemental Figure S13). Indeed, in aqueous medium, S-BT spontaneously undergoes an intramolecular rearrangement to O-BT probably as a result of acyl-migration from sulfur to oxygen [60]. O-BT is then spontaneously converted to thiamine. The conversion of S-BT to O-BT is pH-dependent and proceeds more rapidly at pH 8.5 than at pH 7.0. According to another report, O-BT can be enzymatically converted to thiamine in most tissues [61], however, O-BT did not seem to be more efficient than thiamine in raising tissue thiamine content. In liver, O-BT is partially degraded to thiamine thiazolone, which might be responsible for some toxicity, possibly because of inhibition of TKT1. Species differences concerning the latter reaction are significant. Due to the absence of toxicity of DBT and BFT, it is not likely that large amounts of O-BT are formed.

The metabolization of SuBT differs from that of DBT and BFT as it first requires enzymatic reduction of disulfide-type thiamine derivatives by GSH to yield an open thiol form [62] (Figure 18). Hence, it is remarkable that SuBT has antioxidant properties while its metabolization presumably consumes reduced GSH. However, it has poor anti-inflammatory effects in BV2 cells compared to DBT (Figure 11). The metabolism of S-BT requires the transit through, a most probably very short-lived, open thiol form (ThSD) [63] (Figure 18). We found at best traces of this intermediate after treatment with BFT, DBT, or thiamin-d3 in agreement with the supposedly short-lived character of this intermediate, which is spontaneously formed only slightly (pH < 10) in alkaline medium [64]. It can however not be excluded that, in the presence of oxidants (such as G-S-S-G), the open intermediate may react to form -S-S- disulfides with free cysteine thiols of proteins. This might be an interesting way of post-translational protein regulation.

Hence the active form of DBT and BFT is still unknown and its identification remains a challenge for future research (Figure 18). The active anti-inflammatory compound could be a specific metabolite of DBT while thiamine or a still unknown metabolite of thiamine might be involved in antioxidant effects.

## 5. Conclusions

In conclusion, the present data show that DBT has pleiotropic effects, acting in at least three ways: as a thiamine precursor (by increasing the coenzyme ThDP to combat thiamine deficiency), as an active antioxidant, and as an anti-inflammatory agent at substantially lower concentrations than BFT. It is nontoxic, with no known side-effects and it acts through two different pathways by boosting antioxidant capacities of the cells and anti-inflammatory responses with properties clearly superior to those of BFT. It could therefore be an attractive alternative to BFT with therapeutic potential in neurodegenerative diseases and stress-induced depressive states.

## Supplementary Materials

The following are available online at https://www.mdpi.com/2227-9059/8/9/361/s1, Supplemental Materials and Methods File S1. Experimental design. Supplemental Materials and Methods File S2. Animals and housing conditions, Generation of FUS-tg mice, Wire test, Drug administration, Tissue collection in FUS-tg mice. Supplemental Materials and Methods File S3. Ultrasound radiation, Swim test, Malondialdehyde (MDA) assay. Supplemental Figure S1: Agarose gel electrophoresis of RT-PCR products for detection of thiamine transporter-1 (SLC19A2, THTR-1). Supplemental Figure S2: Effects of thiamine (Thia) or its synthetic precursors. Supplemental Figure S3: Deuterium-labeled thiamine metabolites after incubation of Neuro2a cells with 50 µM labelled thiamine-d3. Supplemental Figure S4: Dibenzoylthiamine (DBT) does not increase the cell density of Neuro2a cells. Supplemental Figure S5: Effect of O-BT (A) and benzoic acid (BA) (B) and on the cell viability of Neuro2a cells in the presence of PQ (0.25 mM, 24 h). Supplemental Figure S6: Buthionine sulfoximine (BSO) decreases the expression of the catalytic subunit of glutamate cysteine ligase (GCLC) in Neuro2a cells. Supplemental Figure S7: Buthionine sulfoximine (BSO, 25 h) decreases the GSH content in absence or in presence of paraquat (PQ, 0.5 mM, 24 h) in Neuro2a cells. Supplemental Figure S8: Paraquat (PQ) induces a similar decrease in cell viability, GSH and NADPH content in Neuro2a cells. Supplemental Figure S9: DMSO (0.1%, diluent of ML385) does not impact the protective effect of DBT (50 µM, 25 h) against PQ-induced cell death in Neuro2a cells. Supplemental Figure S10: Knockdown of Nrf2 using a small double-stranded interfering RNA. (A) Influence of control and Nrf2 siRNA on the expression of Nrf2. (B) Protective effect of DBT against PQ toxicity. Supplemental Figure S11: Effect of PQ on the survival of Neuro2a and BV2 cells in the presence of thiamine or its precursors. Supplemental Figure S12: Intracellular thiamine (A,C) and ThDP (B,D) content after treatment with thiamine or precursors in BV2 cells for 1 or 25 h. Supplemental Figure S13: Effect of benzoic acid (BA) and O-BT on the production of NO_2_^−^ in BV2 cells in the presence of LPS (1 μg/mL, 24 h). Supplemental Figure S14. Treatment with thiamine or DBT did not change latency of falling in the wire test in wild type mice. Supplemental Figure S15. Administration of DBT but not thiamine ameliorates ultrasound-induced increase of malonaldehyde (MDA) concentration in the hippocampus. Supplemental Figure S16: Effect of N-acetylcysteine (NAC) on the levels of GSH in Neuro2a cells (A) and cell viability of PQ-challenged Neuro2a (B) and the production of NO_2_^−^ in LPS-challenged BV2 cells (C).

## Abbreviations

ALS	amyotrophic lateral sclerosis
ARE	antioxidant response element
BFT	benfotiamine
BSO	buthionine sulfoximine
DBT	dibenzoylthiamine
FBS	fetal bovine serum
GCLC	glutamate cysteine ligase catalytic subunit
GSH	reduced glutathione
LPS	lipopolysaccharides
MTT	3-(4,5-dimethylthiazol-2-yl)-2,5-diphenyl-tetrazolium bromide tetrazolium
O-BT	O-benzoylthiamine
S-BT	S-benzoylthiamine
PQ	paraquat
PT	pyrithiamine
ROS	reactive oxygen species
SuBT	sulbutiamine
ThDP	thiamine diphosphate
ThMP	thiamine monophosphate
TKT1	transketolase
TPK	thiamine pyrophosphokinase
