# Supplementary material for: Dibenzoylthiamine Has Powerful Antioxidant and Anti-Inflammatory Properties in Cultured Cells and in Mouse Models of Stress and Neurodegeneration
Source: Biomedicines. 2020 Sep 18;8(9):361. doi: 10.3390/biomedicines8090361 (PMC7555733; doi:10.3390/biomedicines8090361)
Supplement: Supplementary file 1 [file biomedicines-08-00361-s001.zip › biomedicines-884244-supplementary.docx]

Dibenzoylthiamine has powerful antioxidant and anti-inflammatory properties in cultured cells and in mouse models of stress and neurodegeneration

**Margaux Sambon^1^, Anna Gorlova^2,3^, Alice Demelenne^4^, Judit Alhama-Riba^1,5^, Bernard Coumans^6^, Bernard Lakaye^6^, Pierre Wins^1^, Marianne Fillet^4^, Daniel C. Anthony^7,3^, Tatyana Strekalova^2,3^ and Lucien Bettendorff^1,^***

^1^ Laboratory of Neurophysiology, GIGA-Neurosciences, University of Liège, Liège, Belgium; margaux.sambon@uliege.be (M.S.); juditalhamariba@gmail.com (J.A.-R.); winspierre9@gmail.com (P.W.); L.Bettendorff@uliege.be (L.B.)

^2^ Department of Psychiatry and Neuropsychology, Maastricht University, Maastricht, The Netherlands; anna.gorlova204@gmail.com (A.G.); t.strekalova@maastrichtuniversity.nl (T.S.)

^3^ Sechenov First Moscow State Medical University, Institute of Molecular Medicine Laboratory of Psychiatric Neurobiology and Department of Normal Physiology, Moscow, Russia;

^4^ Laboratory for the Analysis of Medicines, CIRM, Department of Pharmacy, University of Liège, Liège

Belgium; alice.demelenne@uliege.be (A.D.); marianne.fillet@uliege.be (M.F.)

^5^ University of Girona, Faculty of Sciences, Spain;

^6^ Laboratory of Molecular Regulation of Neurogenesis, GIGA-Stem Cell, University of Liège, Liège, Belgium; b.coumans@uliege.be (B.C.); b.lakaye@uliege.be (B.L.)

^7^ Department of Pharmacology, Oxford University, Oxford, UK; daniel.anthony@pharm.ox.ac.uk (D.D.A)

***** Correspondence: L.Bettendorff@uliege.be; Tel.: +32-4-366-5967

**Supplemental Materials and Methods**

**Supplemental Materials and Methods File 1**

|  | ***Experimental conditions*** |  |
| --- | --- | --- |
| ***Figure 2*** | Dibenzoylthiamine (DBT) (1, 10, 50 µM) for 0, 2, 4 or 6 h | Neuro2a cells |
| ***Figure 3*** | Dibenzoylthiamine (50 µM, DBT) for 0, 0.5, 1, 2, 4 or 6 h | Neuro2a cells |
| ***Figure 4*** | Dibenzoylthiamine (50 µM, DBT) for 0, 0.5, 1, 2, 4 or 6 h | No cells |
| ***Figure 10*** | Dibenzoylthiamine (50 µM, 25 h, DBT), thiamine (50 µM, 25 h), paraquat (0.25 mM, 24 h, PQ) | Neuro2a cells |
| ***Figure 13***  ***(A, B)*** | Dibenzoylthiamine (25 µM, 25 h, DBT), thiamine (25 µM, 25 h), pyrithiamine (25 µM, 25 h, PT), paraquat (0.25 mM, 24 h, PQ) | Neuro2a cells |
| ***Figure 13***  ***(D, E)*** | Dibenzoylthiamine (25 µM, 25 h, DBT), thiamine (25 µM, 25 h), pyrithiamine (25 µM, 25 h, PT), lipopolysaccharides (1 µg/ml, 24 h, LPS) | BV2 cells |
| ***Figure 14 A*** | Dibenzoylthiamine (0, 10, 50 mg/kg) for 0, 2, 6 or 12 h | Mice (blood) |
| ***Figure 14 B*** | Dibenzoylthiamine (0, 10, 50 mg/kg, 2 h) | Mice (blood) |
| ***Figure 14 C-D*** | Dibenzoylthiamine (0, 50 mg/kg, 2 h) | Mice (C Liver, D cortex) |
| ***Supplemental figure 2*** | Thiamine (50 µM), benfotiamine (50 µM, BFT), sulbutiamine (25 µM, SuBT), dibenzoylthiamine (50 µM, DBT) for 1 or 25 h  In the latter case the cells were treated with 0.25 mM paraquat (PQ) 1 h after addition of thiamine or the precursors. | Neuro2a cells |
| ***Supplemental figure 12*** | Thiamine (50 µM), benfotiamine (50 µM, BFT), sulbutiamine (25 µM, SuBT), dibenzoylthiamine (50 µM, DBT) for 1 or 25 h  In the latter case the cells were treated with 1 μg/ml lipopolysaccharides (LPS) 1 h after addition of thiamine or the precursors. | BV2 cells |

*Experimental design (Cell viability testing)*

|  | ***Experimental conditions*** | ***Cell type*** |
| --- | --- | --- |
| ***Figure 5 (A/B)*** | Dibenzoylthiamine (DBT) (1, 2, 5, 10, 25, 50 µM, 25 h), paraquat (0.25 mM, 24 h, PQ) | Neuro2a cells |
| ***Figure 6 B*** | Dibenzoylthiamine (50 µM, 25 h, DBT), thiamine (50 µM, 25 h), buthionine sulfoximine (0.5 mM, 25 h, BSO), paraquat (0.25 mM, 24 h, PQ) | Neuro2a cells |
| ***Figure 9 B*** | Dibenzoylthiamine (DBT) (25, 50 µM, 25 h), paraquat (0.25 mM, 24 h, PQ) | 3T3 cells |
| ***Figure 9 C*** | Dibenzoylthiamine (50 µM, 25 h, DBT), thiamine (50 µM, 25 h), ML385 (10 µM, 25 h), paraquat (0.25 mM, 24 h, PQ) | Neuro2a cells |
| ***Figure 13C*** | Dibenzoylthiamine (25 µM, 25 h, DBT), thiamine (25 µM, 25 h), pyrithiamine (25 µM, 25 h), paraquat (0.25 mM, 24 h, PQ) | Neuro2a cells |
| ***Supplemental figure 4*** | Dibenzoylthiamine (50 µM, 25 h, DBT), thiamine (50 µM, 25 h) | Neuro2a cells |
| ***Supplemental figure 5*** | O-benzoylthiamine (O-BT) (10, 25, 50 µM, 25 h), benzoic acid (BA) (25, 50, 100 µM, 25 h), paraquat (0.25 mM, 24 h, PQ) | Neuro2a cells |
| ***Supplemental figure 9*** | Dibenzoylthiamine (50 µM, 25 h, DBT), thiamine (50 µM, 25 h), dimethyl sulfoxide (0.1%, 25 h, DMSO), paraquat (0.25 mM, 24 h, PQ) | Neuro2a cells |
| ***Supplemental figure 10*** | Dibenzoylthiamine (50 µM, 25 h, DBT), thiamine (50 µM, 25 h), Nrf2- control siRNA (50 nM, 24-48 h), paraquat (0.25 mM, 24 h, PQ) | Neuro2a cells |
| ***Supplemental figure 11*** | Thiamine (50 µM, 25 h), benfotiamine (50 µM, 25 h, BFT), sulbutiamine (25 µM, 25 h, SuBT), dibenzoylthiamine (50 µM, 25 h, DBT), paraquat (0.25 mM, 24 h, PQ) | Neuro2a cells (A)  BV2 cells (B) |
| ***Supplemental figure 16*** | N-acetyl-L-cysteine (50 µM, 5 mM, 25 h, NAC), paraquat (0.25 mM, 24 h, PQ) | Neuro2a cells |

*Experimental design (Western blot)*

|  | ***Experimental conditions*** | ***Antibody*** | ***Cell type*** |
| --- | --- | --- | --- |
| ***Figure 6 C-D*** | Thiamine (50 µM, 25 h), benfotiamine (50 µM, 25 h, BFT), sulbutiamine (25 µM, 25 h, SuBT), dibenzoylthiamine (50 µM, 25 h, DBT), paraquat (0.25 mM, 24 h, PQ) | Anti-GCLC  Anti-α-tubulin | Neuro2a cells |
| ***Figure 11 D*** | Thiamine (50 µM, 25 h), benfotiamine (50 µM, 25 h, BFT), sulbutiamine (25 µM, 25 h, SuBT), dibenzoylthiamine (50 µM, 25 h, DBT), lipopolysaccharides (1 µg/ml, 24 h, LPS) | Anti-iNOS  Anti-α-tubulin | BV2 cells |
| ***Figure 11 E*** | Dibenzoylthiamine (DBT) (1, 10, 25, 50 µM, 25 h), lipopolysaccharides (1 µg/ml, 24 h, LPS) | Anti-iNOS  Anti-α-tubulin | BV2 cells |
| ***Figure 12*** | Thiamine (50 µM, 2 h), benfotiamine (50 µM, 2 h, BFT), sulbutiamine (25 µM, 2 h, SuBT), dibenzoylthiamine (50 µM, 2 h, DBT), lipopolysaccharides (1 µg/ml, 1 h, LPS) | Anti-p65  Anti-α-tubulin  Anti-lamin B1 | BV2 cells |
| ***Supplemental figure 6*** | Buthionine sulfoximine (0.1, 0.5, 1, 5 mM, 6 h, BSO), paraquat (0.25 mM, 6 h, PQ) | Anti-GCLC  Anti-α-tubulin | Neuro2a cells |
| ***Supplemental figure 10*** | Dibenzoylthiamine (50 µM, 25 h, DBT), thiamine (50 µM, 25 h), Nrf2- control siRNA (50 nM, 24-48 h), paraquat (0.25 mM, 24 h, PQ) | Anti-Nrf2  Anti-α-tubulin | Neuro2a cells |

*Experimental design (Nitric oxide assay)*

|  | ***Experimental conditions*** |
| --- | --- |
| ***Figure 11 A*** | Thiamine (50 µM, 25 h), benfotiamine (50 µM, 25 h, BFT), sulbutiamine (25 µM, 25 h, SuBT), dibenzoylthiamine (50 µM, 25 h, DBT), lipopolysaccharides (1 µg/ml, 24 h, LPS) |
| ***Figure 11 B*** | Thiamine (25 h), benfotiamine (25 h, BFT), sulbutiamine (25 h, SuBT), dibenzoylthiamine (25 h, DBT), lipopolysaccharides (1 µg/ml, 24 h, LPS) |
| ***Figure 13 F*** | Dibenzoylthiamine (25 µM, 25 h, DBT), pyrithiamine (25 µM, 25 h, PT), lipopolysaccharides (1 µg/ml, 24 h, LPS) |
| ***Supplemental figure 13 A*** | Benzoic acid (25, 50, 100 µM, 25 h, BA), lipopolysaccharides (1 µg/ml, 24 h, LPS) |
| ***Supplemental figure 13 B*** | O-benzoylthiamine (10, 25, 50 µM, 25 h, O-BT), lipopolysaccharides (1 µg/ml, 24 h, LPS) |
| ***Supplemental figure 13 C*** | Dibenzoylthiamine (25 µM, 25 h, DBT), benzoic acid (25 µM, 25 h, BA), lipopolysaccharides (1 µg/ml, 24 h, LPS) |
| ***Supplemental figure 16 C*** | N-acetyl-L-cysteine (50 µM, 5 mM, 25 h), lipopolysaccharides (1 µg/ml, 24 h, LPS) |

**Supplemental Materials and Methods File 2**

*Animals and* *housing conditions*

FUS[1–359]-transgenic female (FUS-tg) and their wild type littermates were provided by the FDA-certified IPAC RAS facilities, Chernogolovka, Moscow region (http://www.ipac.ac.ru/index.html). BALB/c male mice were obtained from certified Charles River provider, Stolbovaja, RAS, Moscow region (http://www.spf-animals.ru/about/providers/animals). Male mice were single housed, and female mice were housed 2–3 per cage under standard conditions (22±1°C, 55% humidity, reversed 12-h light/dark cycle with lights on at 19:00, food and water *ad libitum*). Experimental procedures were set up in accordance with a Directive 2010/63/EU and approved by the local veterinarian Committee for Bioethics of IPAC RAS (N19-16.06.2017) and MSMU (22/10/17-MSMU-35).

*Generation of FUS-tg mice*

Generation of FUS-tg mice was performed as described elsewhere (Shelkovnikova et al., 2013). Briefly, a fragment of human FUS [1–359] cDNA including 9 bp of 5′-UTR was cloned into Thy-1 promoter plasmid 323-pTSC21k. A gel-purified fragment obtained by digestion of the resulting plasmid DNA with NotI was used for microinjection of mouse oocytes. Transgenic animals were identified by PCR analysis of DNA from ear biopsies by the presence of 255-bp product (primers 5′-TCTTTGTGCAAGGCCTGGGT-3′ and 5′-AGAAGCAAGACCTCTGCAGAG-3′). The original transgenic line on C57Bl6/CBA genetic background was backcrossed with CD1 wild type mice for several (>7) generations. About 15 animals per group were used.

*Wire test*

FUS-tg male mice and their wild type littermates were allowed to grip a horizontal wire (diameter 0.3 cm, height above the surface 60 cm) for 180 seconds. The latency of falling was recorded as described elsewhere (de Munter et al., 2020; Veniaminova et al., 2020).

*Drug administration*

Experimental solutions replaced normal drinking water. Thiamine, DBT (Sigma-Aldrich, St. Louis, MO, USA) and riluzole (Sandoz, Almere, Netherlands) were dissolved in tap water and changed every 4-5 days as described elsewhere (Markova al., 2017; Vignisse et al., 2017; de Munter et al., 2020). Celecoxib-containing food pellets were produced as described elsewhere (Costa-Nunes et al., 2015; de Munter et al., 2020) and replaced standard laboratory diet. Concentrations of treatments were adjusted to the daily liquid or food intake. Drinking behavior and diet consumption were monitored by evaluating the 24-h liquid and food intake during the first three days of dosing. No group differences in these measures were found suggesting normal drinking behavior in ultrasound-exposed and dosed mice, as well as normal feeding behavior in celecoxib-treated mice.

*Tissue collection in FUS-tg mice*

At the age of fifteen weeks, non-treated female FUS-tg and wild type mice were anaesthetized by sodium pentobarbitone and perfused with NaCl. Lumbar parts of the spinal cord were collected as described elsewhere (de Munter et al., 2020) and immediately frozen at −80 °C on a dry ice for a subsequent malondialdehyde (MDA) assay (*see below*).

**Supplemental Materials and Methods File** **3**

*Ultrasound radiation*

Experimental groups of BALB/c mice were subjected to the continuous ultrasound emission of average intensity of 50±5dB and frequencies in a 20-45Hz range delivered via a manufactured device (Weitech, Wavre, Belgium) using a random schedule of alternating frequencies as described elsewhere (Pavlov et al., 2019; Gorlova et al., 2019). During the 10-min periods, ultrasound frequencies fluctuated at short time spans of ≤1 s. 35% of the emission time consisted of 20-25 and 25-40 kHz frequencies, and 30% of the emission time consisted of 40-45 kHz frequencies. The ultrasound device was suspended 2 m above the cages of the experimental groups with an average horizontal distance to cages of 2.5 m. The position of the cages with respect to the stimulator was changed weekly. The selectivity of the adverse effects of low-frequency ultrasound during the emission period in comparison with the potential general negative effects of a constant noise described here was demonstrated previously (Morozova et al., 2016; Gorlova et al., 2019).

*Swim test*

This test was carried out as described previously (Malatynska et al., 2012; Strekalova et al., 2015; Pavlov et al., 2017). BALB/c mice were placed into a plastic transparent pool (diameter of 15 cm and a height of 25 cm) filled with water to the depth for 17 cm; water temperature was +2 3°C, light intensity was 15 Lux. Total duration of floating behavior, defined by the absence of any directed movements of animals’ head and body, was scored offline during the 6 min-period using Any-maze software (Stoelting Co, Wood Dale, IL, USA).

*Malondialdehyde (MDA) assay*

MDA content was measured following Abcam ab118970 kit instructions (Abcam, Eugene, OR, USA). Briefly, the tissue was washed in cold PBS and homogenized in lysis solution, and then centrifuged at 13000 g for 10 min. TBA reagent was added to supernatant and, after incubation at 95 °C for 60 min and cooling to room temperature in an ice bath, supernatant was analyzed at 532nm in a 96-well microplate as described elsewhere (Costa-Nunes et al., 2020).

**References for supplemental Materials and Methods**

Costa-Nunes J.P., Cline B.H., Araújo-Correia M., Valença A., Markova N., Dolgov O., Kubatiev A., Yeritsyan N., Steinbusch H.W., Strekalova T. Animal Models of Depression and Drug Delivery with Food as an Effective Dosing Method: Evidences from Studies with Celecoxib and Dicholine Succinate. Biomed Res Int. 2015: 596126.

Costa-Nunes J.P., Gorlova A., Pavlov D., Cespuglio R., Gorovaya A., Proshin A., Umriukhin A., Ponomarev E., Kalueff A., Strekalova T., Schroeter C. Ultrasound stress compromises the correlates of emotional-like states and brain AMPAR expression in mice: effects of antioxidant and anti-inflammatory herbal treatment. Stress. 2020, 13: 1-15.

de Munter J.P.J.M., Shafarevich I., Liundup A., Pavlov D., Wolters E.C., Gorlova A., Veniaminova E., Umriukhin A., Kalueff A., Svistunov A., Kramer B.W., Lesch K.P., Strekalova T. Neuro-Cells therapy improves motor outcomes and suppresses inflammation during experimental syndrome of amyotrophic lateral sclerosis in mice. CNS Neurosci Ther. 2020, 26: 504-517.

Gorlova A., Pavlov D., Anthony D.C., Ponomarev E.D., Sambon M., Proshin A., Shafarevich I., Babaevskaya D., Lesсh K.P., Bettendorff L., Strekalova T. Thiamine and benfotiamine counteract ultrasound-induced aggression, normalize AMPA receptor expression and plasticity markers, and reduce oxidative stress in mice. Neuropharmacology. 2019, 156: 107543.

Malatynska E., Steinbusch H.W., Redkozubova O., Bolkunov A., Kubatiev A., Yeritsyan N.B., Vignisse J., Bachurin S., Strekalova T. Anhedonic-like traits and lack of affective deficits in 18-month-old C57BL/6 mice: implications for modeling elderly depression. Exp. Gerontol. 2012, 47: 552–564.

Markova N., Bazhenova N., Anthony D.C., Vignisse J., Svistunov A., Lesch K.P., Bettendorff L., Strekalova T. Thiamine and benfotiamine improve cognition and ameliorate GSK-3β- associated sterss-induced behaviours in mice. Prog Neuro-Psychopharmacol Biol Psychiatry. 2017, 75: 148–156.

Morozova A., Zubkov E., Strekalova T., Kekelidze Z., Storozeva Z., Schroeter C.A., Bazhenova N., Lesch K.P., Cline B.H., Chekhonin V. Ultrasound of alternating frequencies and variable emotional impact evokes depressive syndrome in mice and rats. Prog Neuro-Psychopharmacol Biol Psychiatry. 2016, 68, 52–63.

Pavlov D., Bettendorff L., Gorlova A., Olkhovik A., Kalueff A.V., Ponomarev E.D., Inozemtsev A., Chekhonin V., Lesсh K.P., Anthony D.C., Strekalova T. Neuroinflammation and aberrant hippocampal plasticity in a mouse model of emotional stress evoked by exposure to ultrasound of alternating frequencies. Prog Neuropsychopharmacol Biol Psychiatry. 2016, 90: 104-116.

Pavlov D., Markova N., Bettendorff L., Chekhonin V., Pomytkin I., Lioudyno V., Svistunov A., Ponomarev E., Lesch K.P., Strekalova T. Elucidating the functions of brain GSK3α: Possible synergy with GSK3β upregulation and reversal by antidepressant treatment in a mouse model of depressive-like behaviour. Behav Brain Res. 2017, 335: 122-127.

Shelkovnikova Tatyana A., Peters Owen M., Deykin Alexey V., Connor-Robson Natalie, Robinson Hannah, Ustyugov Alexey A., Bachurin Sergey O., Ermolkevich Tatyana G., Goldman Igor L., Sadchikova Elena R., Kovrazhkina Elena A., Skvortsova Veronica I., Ling Shuo-Chien, Cruz Sandrine Da, Parone Philippe A., Buchman Vladimir L., Ninkina Natalia N. Fused in Sarcoma (FUS) Protein Lacking Nuclear Localization Signal (NLS) and Major RNA Binding Motifs Triggers Proteinopathy and Severe Motor Phenotype in Transgenic Mice. J. Biol. Chem. 2013, 288(35): 25266-74. doi: 10.1074/jbc.M113.492017.

Strekalova Tatyana, Evans Matthew, Chernopiatk Anton, Couch Yvonne, Costa-Nunes João, Cespuglio Raymond, Chesson Lesley, Vignisse Julie, Steinbusch Harry W, Anthony Daniel C, Pomytkin Igor, Lesch Klaus-Peter. Deuterium Content of Water Increases Depression Susceptibility: The Potential Role of a Serotonin-Related Mechanism. Behav. Brain Res. 2015, 277: 237-44. doi: 10.1016/j.bbr.2014.07.039.

**Supplemental Figures**


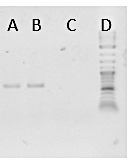


1000

900

800

700

600

500

400

300

200

100

3000

2000

1500

1200

**Supplemental figure 1: Agarose gel electrophoresis of RT-PCR products for detection of thiamine transporter-1 (SLC19A2, THTR-1).** (A) Neuro2a cells (DMEM), (B) Neuro2a cells (DMEM thiamin-restricted), (C) H_2_O as negative control and (D) 100 bp DNA Ladder (the size of the markers is indicated, Quick-Load Purple 2-Log DNA ladder, New England Biolabs). All PCR products showed a band at position 610 bp in agreement with the expected amplicon size.

| A   | 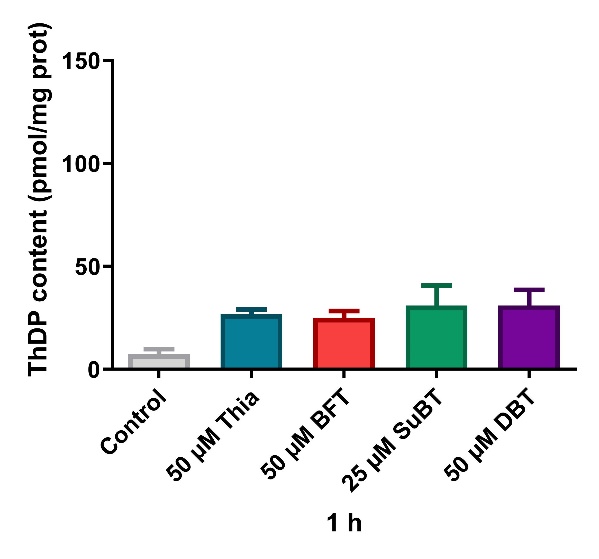B |
| --- | --- |
| 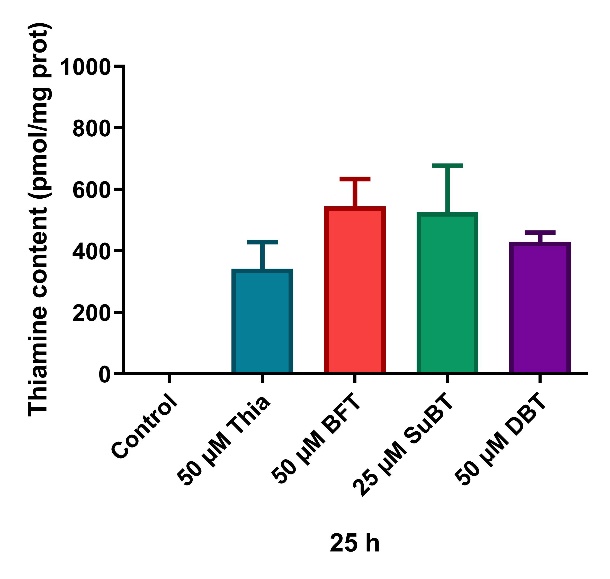C | 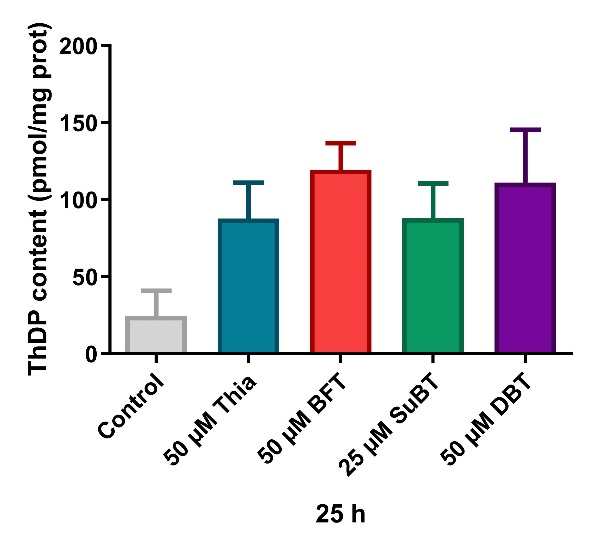D |

**Supplemental figure 2: Effects of thiamine (Thia) or its synthetic precursors (benfotiamine, BFT; sulbutiamine, SuBT and dibenzoylthiamine, DBT) on thiamine (A, C) and ThDP (B, D) content in Neuro2a cells after 1 h (A, B) or 25 h (C, D).** The cells were grown in DMEM thiamine-restricted medium as described in Materials and Methods. At t=0, thiamine, BFT, SuBT or DBT were added at the concentrations indicated. Intracellular thiamine and ThDP contents were analyzed by HPLC after 1 and 25 h. In the latter case the cells were treated with 0.25 mM PQ 1 h after addition of thiamine or the precursors. Control thiamine levels are very low (1 pmol/mg of protein) compared to the levels in the presence of precursors (see figure 2). The results are expressed as mean ± SD (n=3).

**
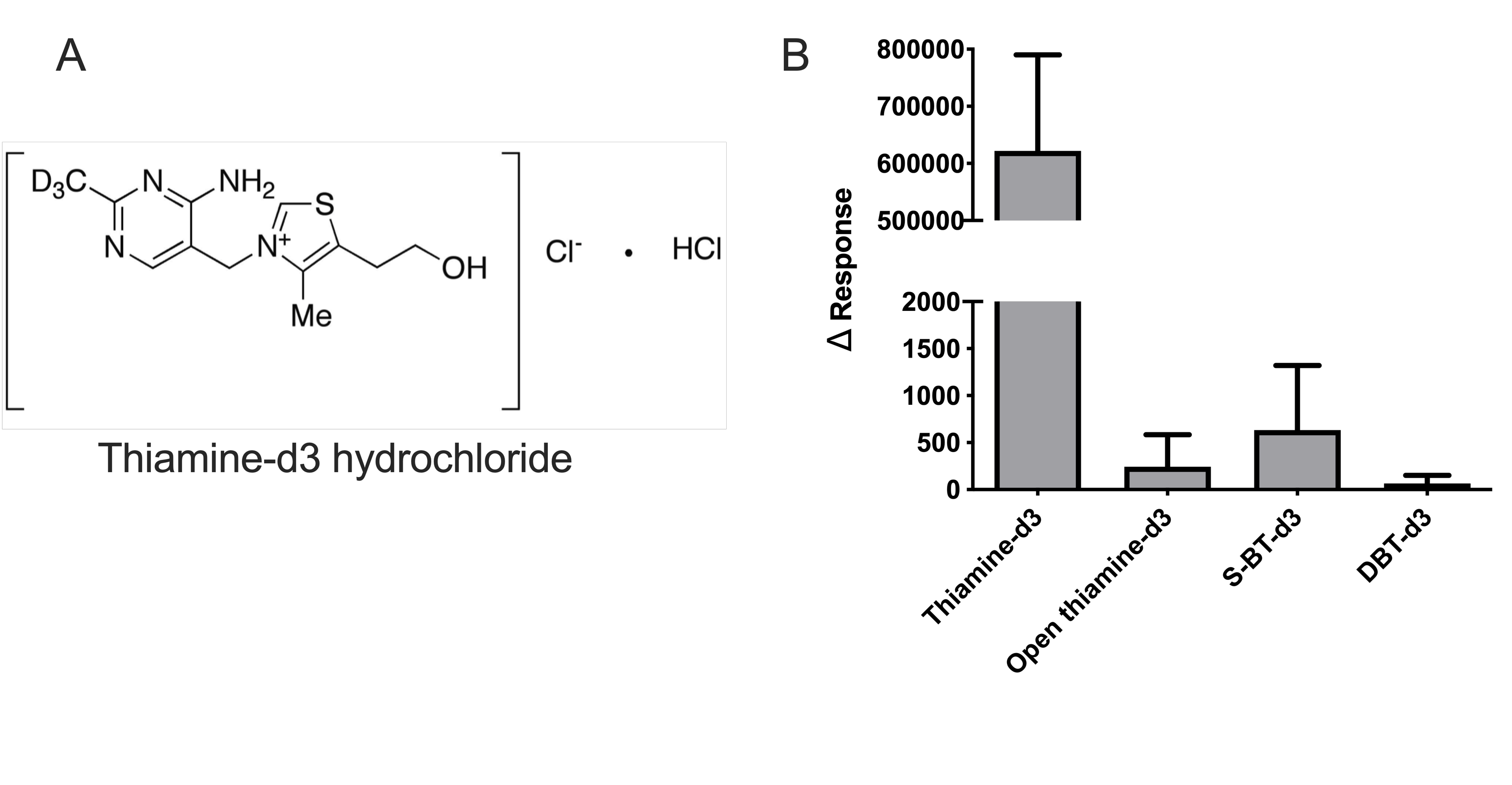
**

**Supplemental figure 3: Deuterium-labeled thiamine metabolites after incubation of Neuro2a cells with 50 µM labelled thiamine-d3.** (A) Structural formula of thiamine-3d. (B) Difference between the responses without and with incubation of the cells for 1 h with thiamine-d3. The open form of thiamine-d3, S-BT-d3 and DBT-d3 could be detected in very low quantities. The response of the compounds in the cells incubated with thiamine d3 were subtracted by the response of the compounds in the cells used as negative control. (mean ± SD, n = 12)

|  |
| --- |

| 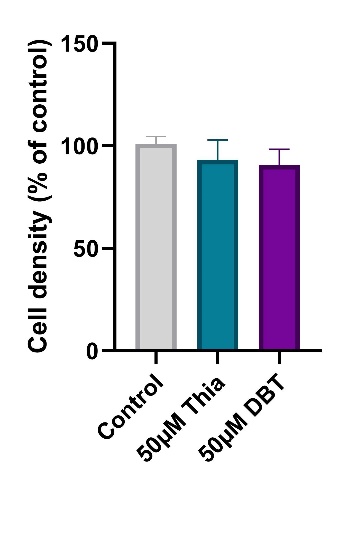 | **Supplemental figure 4: DBT does not increase the cell density of a Neuro2a cells culture in the absence of PQ.** Thiamine and DBT at 50 µM were added to cells and the cell density (MTT test) was tested 25 h later. The results are expressed as mean ± SD (n=3). Statistical analysis was made by one-way ANOVA. | |
| --- | --- | --- |
| \| A 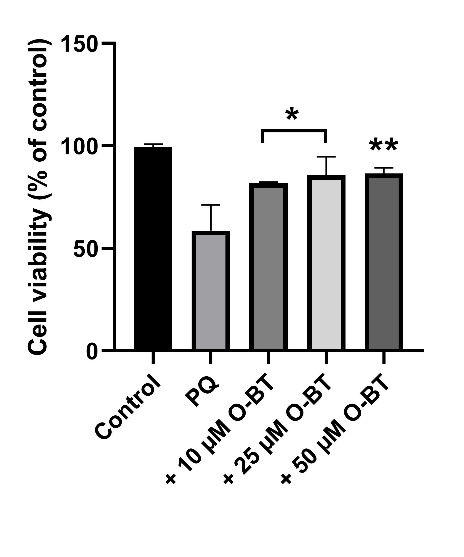 \| B  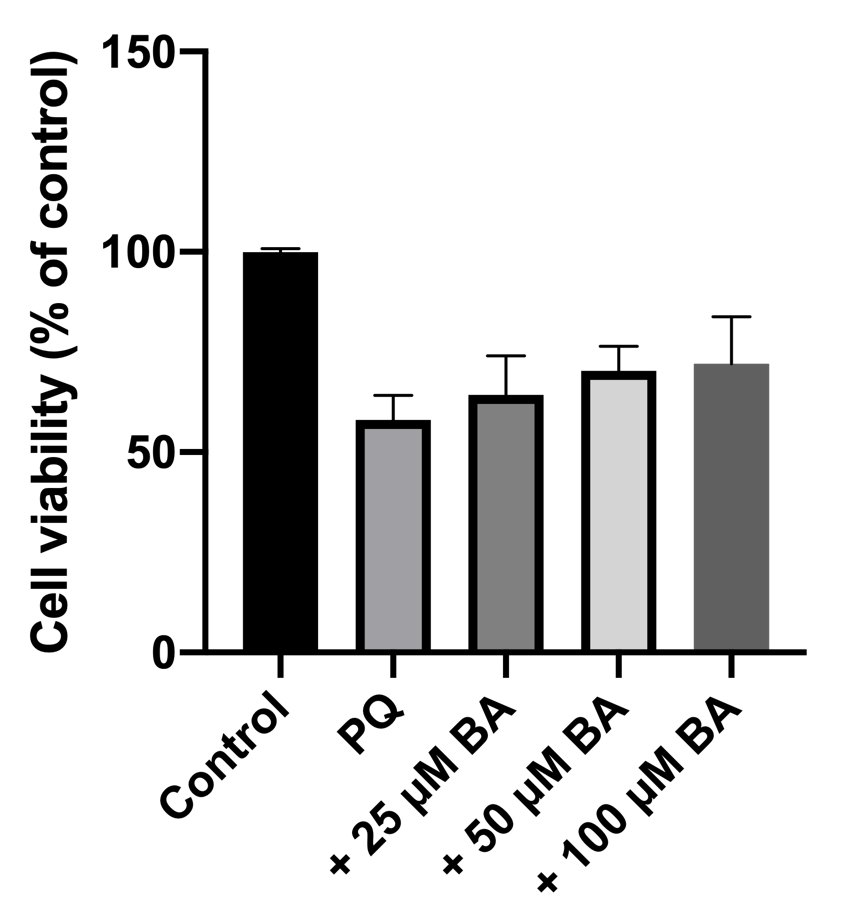 \| \| --- \| --- \|   **Supplemental figure 5: Effect of O-benzoylthiamine (O-BT) (A) and benzoic acid (BA) (B) and on the cell viability of Neuro2a cells in the presence of PQ (0.25 mM, 24 h**). The data are expressed as mean ± SD (n=3-5, ***, p<0.001, ANOVA followed by a Tukey post-hoc test). | | |
| 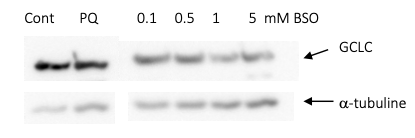 | | **Supplemental figure 6:** **Buthionine sulfoximine (BSO) prevents the expression of the catalytic subunit of glutamate cysteine ligase (GCLC) in Neuro2a cells under control conditions.** The cells were incubated in the presence of BSO for 6 h. PQ does not affect GCLC expressions (immunoblot after SDS-PAGE). |

| 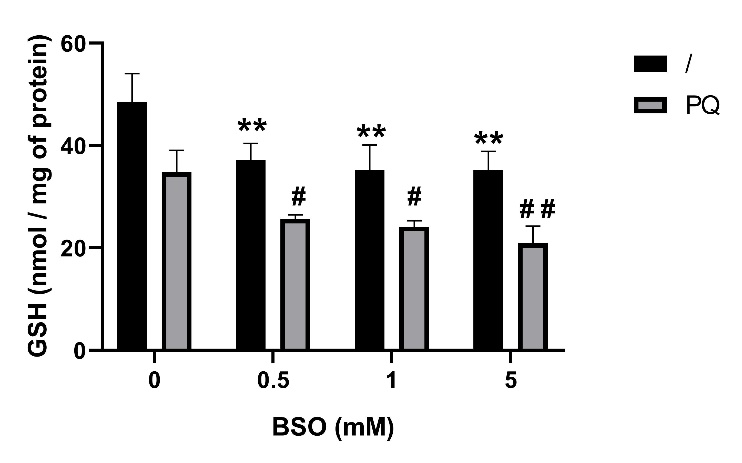 | **Supplemental figure 7:** **Buthionine sulfoximine (BSO, 25 h) decreases the GSH content in absence or in presence of paraquat (PQ, 0.5 mM, 24 h) in Neuro2a cells.** The results are expressed as mean ± SD (n=3). Statistical analysis was made by two-way ANOVA followed by Tukey multiple comparisons test (**, p<0.01 vs condition control, # p<0.05 vs conditon PQ, ##, p<0.01 vs condition PQ) . | |
| --- | --- | --- |
| 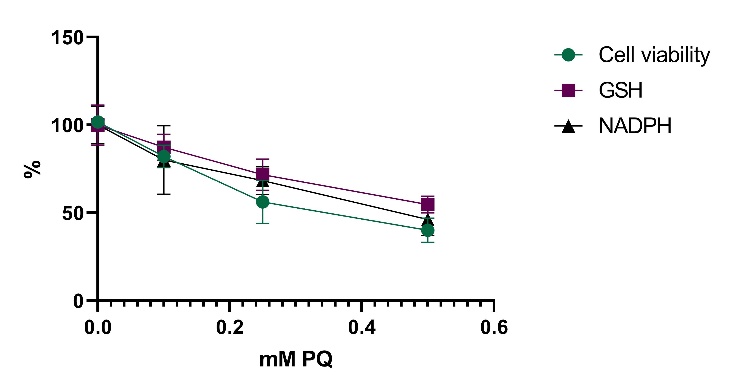 | | **Supplemental figure 8: Paraquat (PQ) induces a similar decrease in cell viability, GSH and NADPH content in Neuro2a cells.** The results are expressed as mean ± SD (n=3). |

| 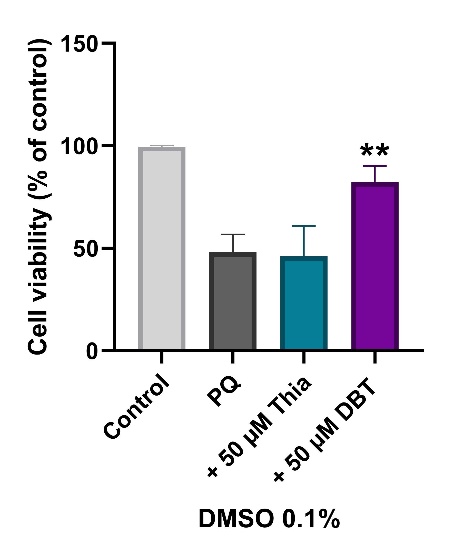 | **Supplemental figure 9: DMSO (0.1%, diluent of ML385) does not impact the protective effect of DBT (50 µM, 25 h) against PQ-induced cell death in Neuro2a cells.** The results are expressed as mean ± SD (n=4). Statistical analyses was made by one-way ANOVA followed by Tukey multiple comparisons test (**, p<0.01 vs condition PQ) |
| --- | --- |

| A  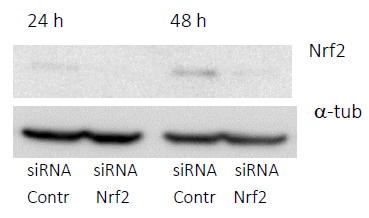 | B  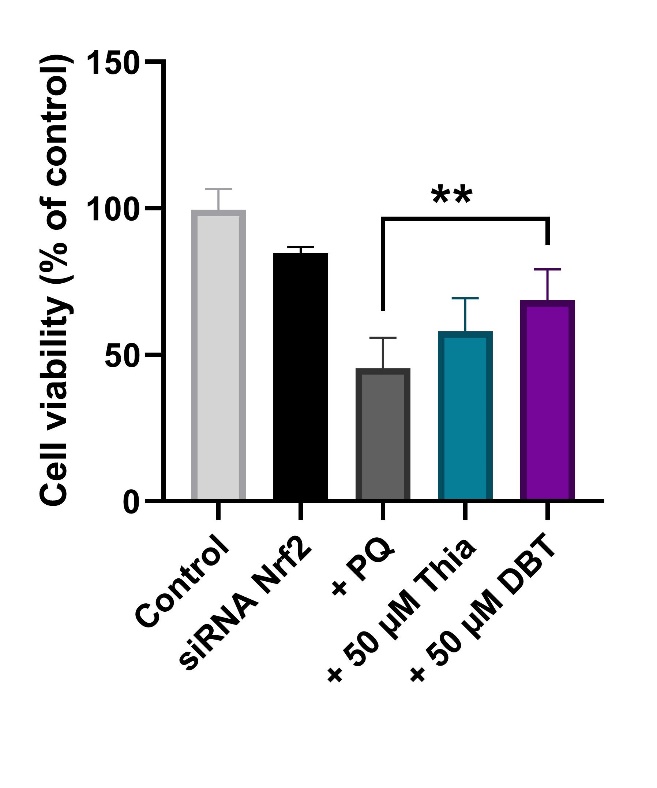 |
| --- | --- |

**Supplemental figure 10: Knockdown of Nrf2 using a small double-stranded interfering RNA (siRNA).** (A) Influence of control and Nrf2 siRNA on the expression of Nrf2 after 24 h and 48 h (immunoblot after SDS-PAGE). At 48 h, the cells were treated with paraquat (0.25 mM). (B) Impact of Nrf2 siRNA on the protective effect of DBT (50 μM, 25 h) against PQ-induced cell death in Neuro2a cells. The results are expressed as mean ± SD (n=5). Statistical analysis was made by one-way ANOVA followed by Tukey multiple comparisons test (** p < 0.01).

| **Neuro2a cells**  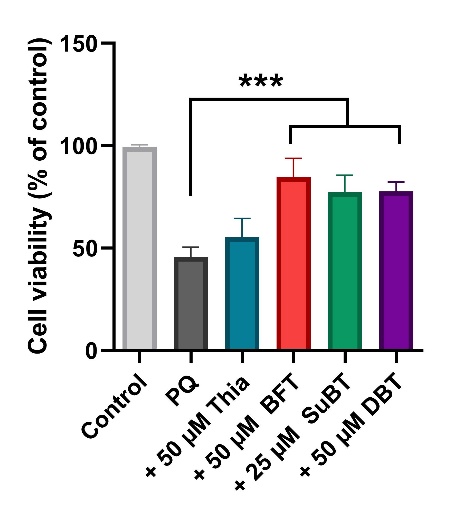 | **BV2 cells**  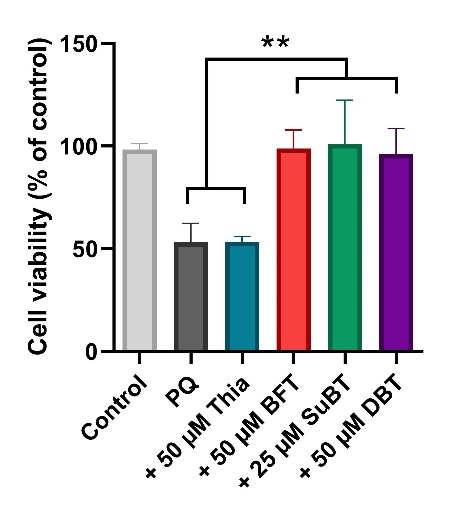 |
| --- | --- |

**Supplemental figure 11: Effect of PQ on the survival of Neuro2a and BV2 cells in the presence of thiamine or its precursors.** The cells were incubated for 25 hours in the absence (control) or presence of thiamine (Thia, 50 μM) or its prodrugs (BFT, 50 μM ; DBT, 50 μM or SuBT, 25 μM). PQ (0.25 mM) was added after 1 hour and the survival rate was determined 24 hours later. The data are expressed as mean ± SD (n=3, **, p<0.01, ***, p<0.001, one-way ANOVA followed by Tukey’s post-hoc test).

| A  **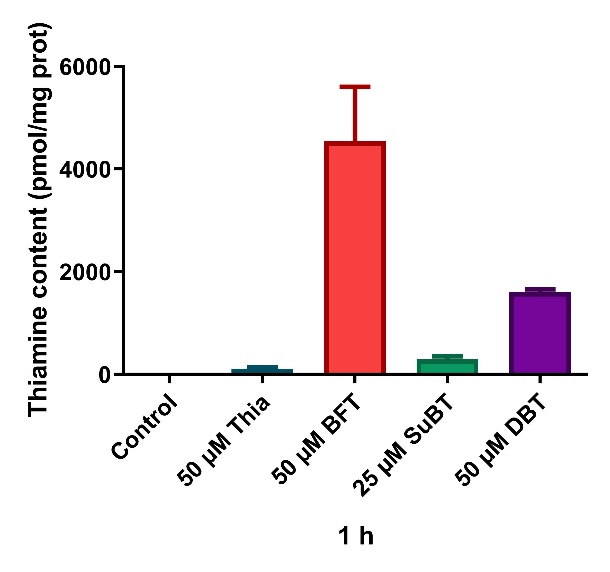** | B  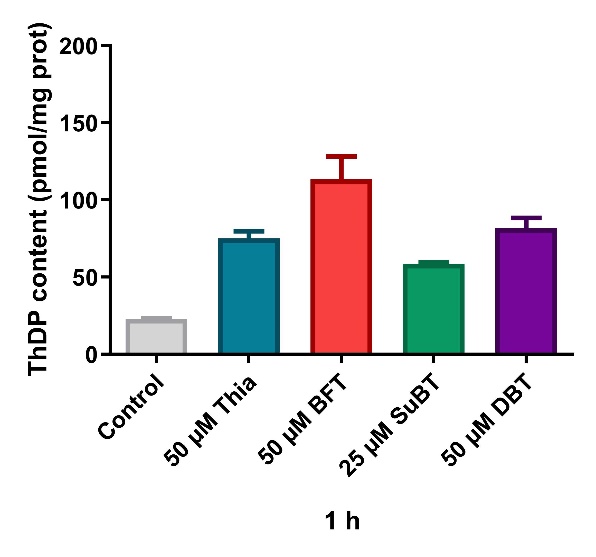 |
| --- | --- |
| C  **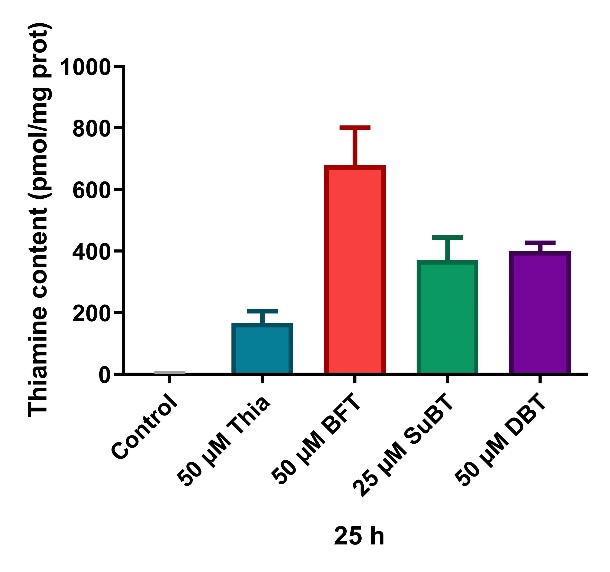** | D  **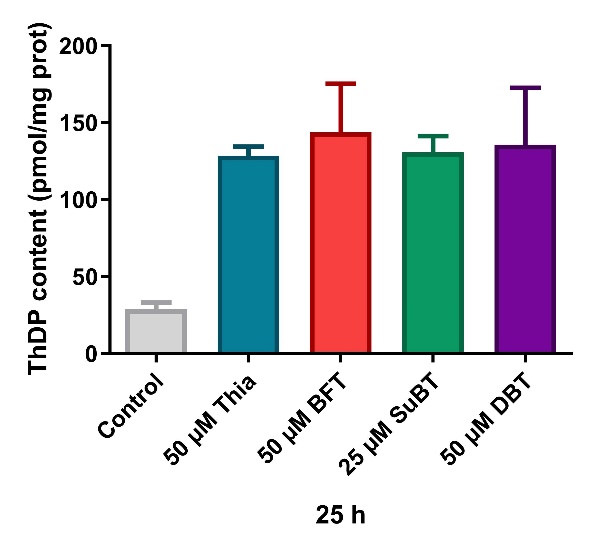** |
| **Supplemental figure 12: Intracellular thiamine (A, C) and ThDP (B, D) content after treatment with thiamine or prodrugs in BV2 cells for 1 or 25 hours.** In the latter case the cells were treated with 1 μg/ml LPS 1 h after addition of thiamine or the precursors. The protocol was as described in legend to supplemental figure 2. (mean ± SD, n=3) | |

| A  **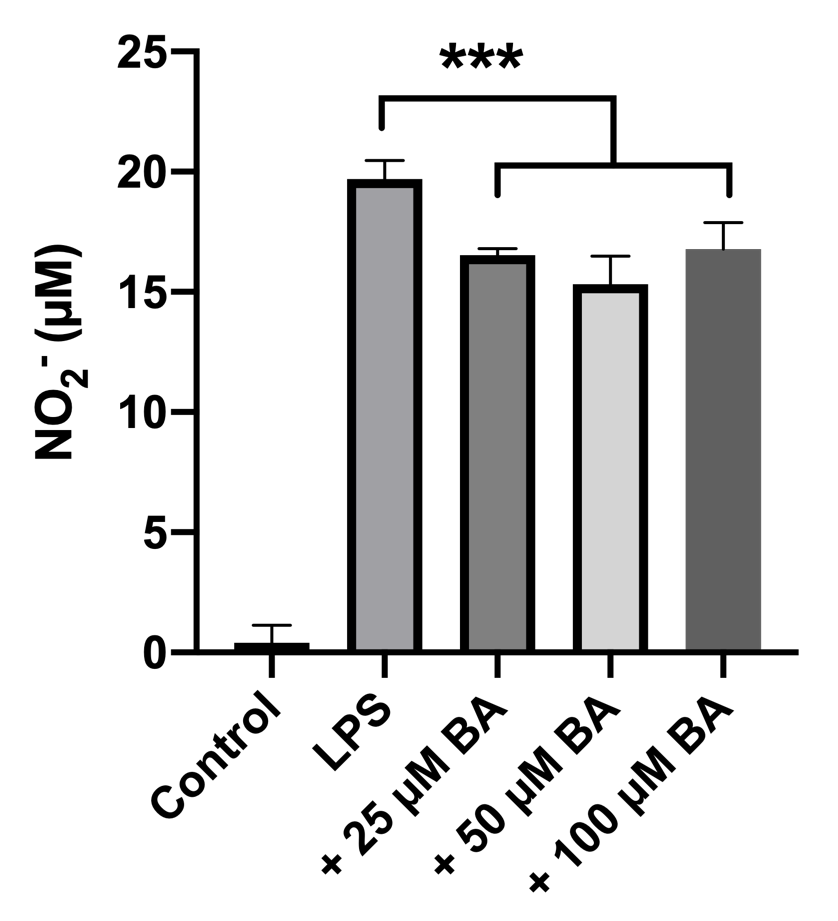** | B  **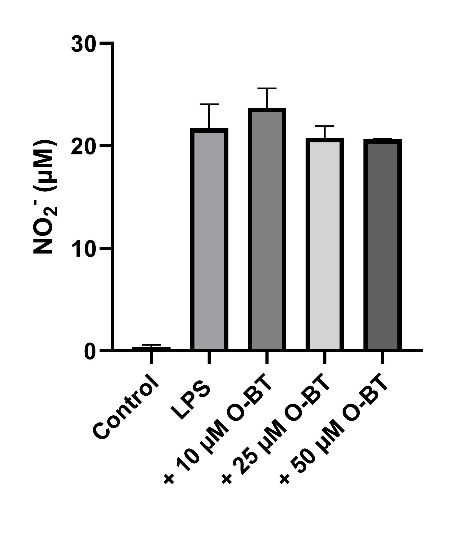** | C  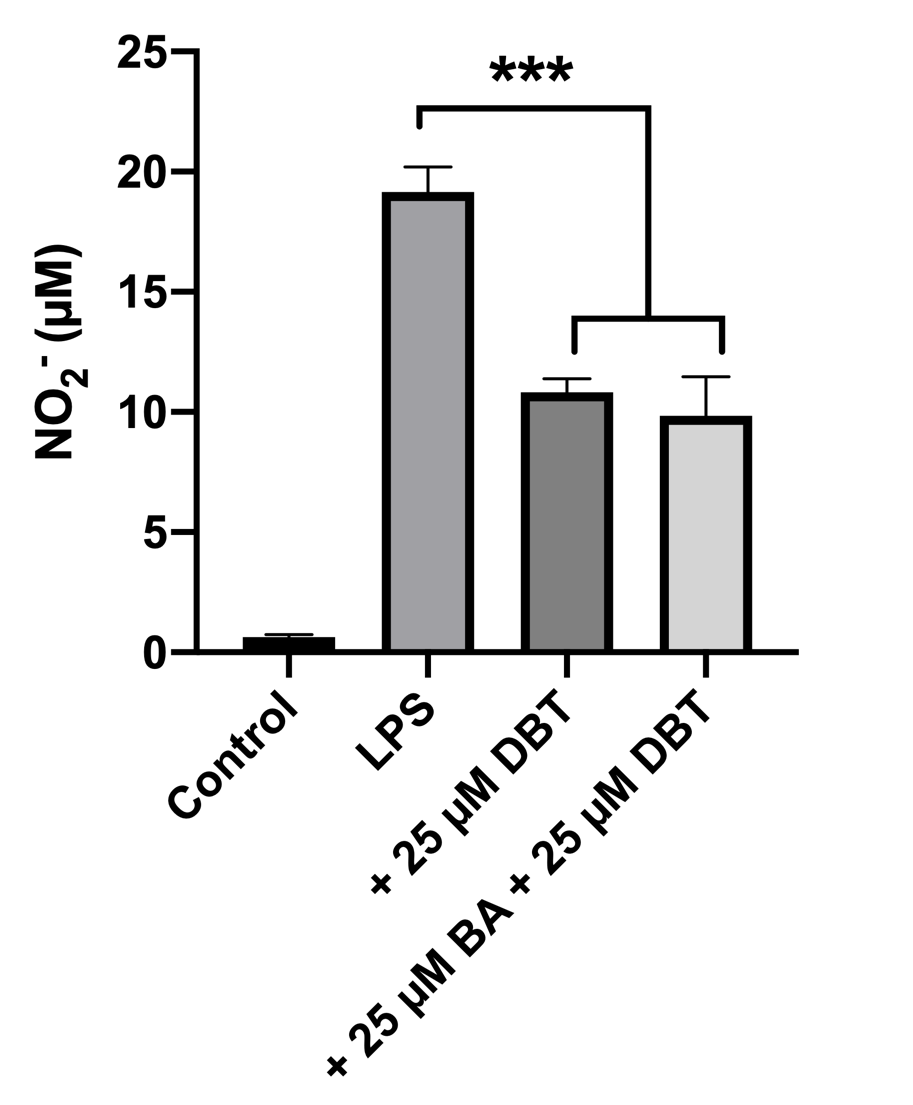 |
| --- | --- | --- |

| **Supplemental figure 13: Effect of benzoic acid (BA) and O-benzoylthiamine (O-BT) on the production of NO^-^_2_ in BV2 cells in the presence of LPS (1 μg/ml, 24 h**). (A) BA. (B) O-BT. (C) Combined effects of benzoic acid and DBT. The data are expressed a mean ± SD (n=3-5, ***, p<0.001, ANOVA followed by a Tukey post-hoc test). |
| --- |


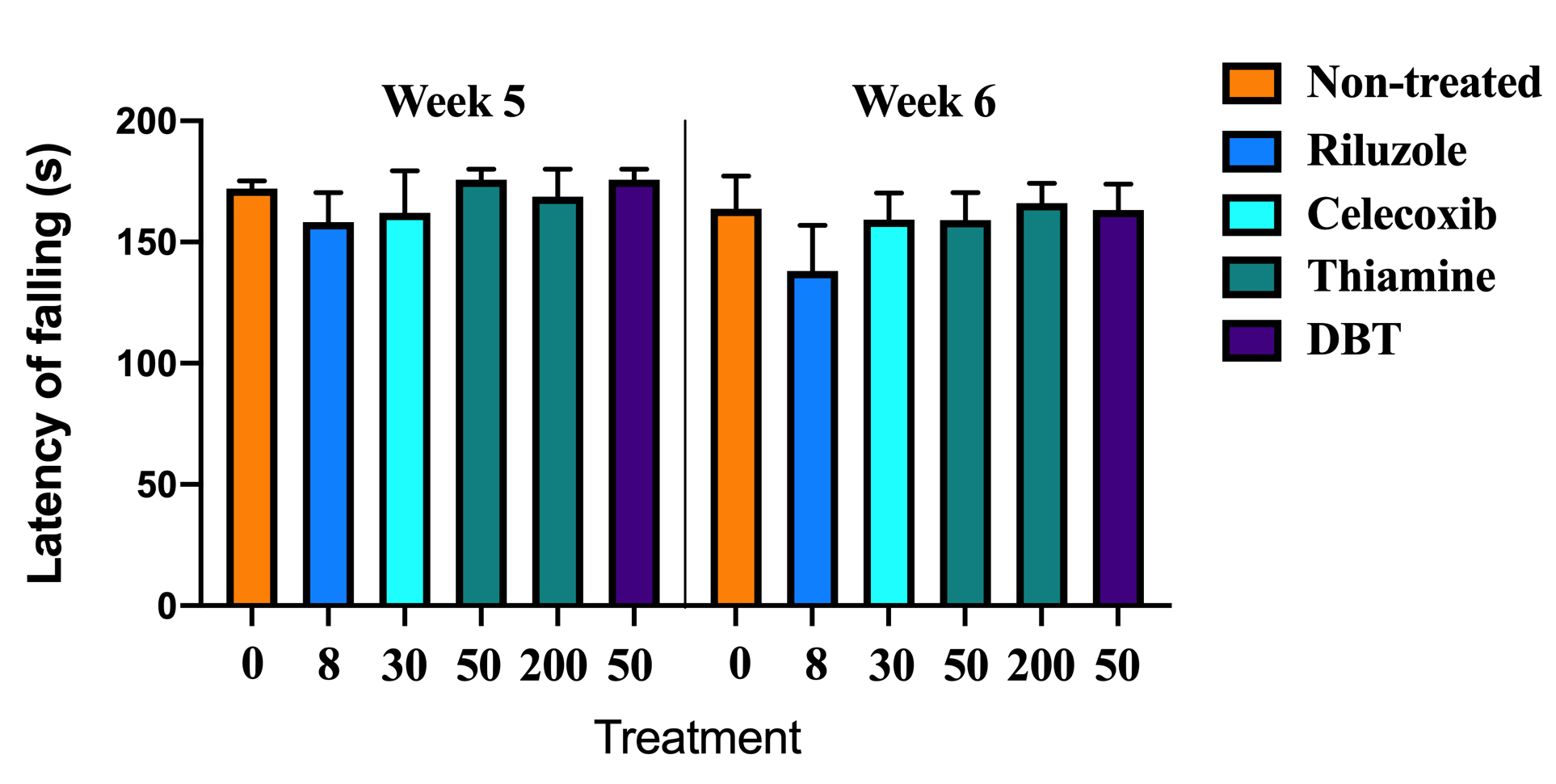


**Supplemental Figure 14: Treatment with thiamine or DBT did not change latency of falling in the wire test in wild type mice.** No significant differences were found between groups on week 5 and week 6 of the study, p>0.05, two-way ANOVA. Doses are expressed in mg/kg per day. DBT = dibenzoyl thiamine. 7-16 animals per group were used. Bars are mean ± SEM.

**Supplemental Figure 15: Administration of DBT but not thiamine ameliorates ultrasound-induced increase of malonaldehyde (MDA) concentration in the hippocampus.** Ultrasound-exposed vehicle-treated mice displayed a significant elevation of the MDA concentration in the hippocampus compared to control group. Ultrasound-exposed groups treated with DBT at the doses of 25 or 200 mg/kg per day showed significantly decreased MDA concentration than vehicle-treated mice exposed to the ultrasound stress (*, p<0.05 vs. control mice, #, p<0.05 vs. vehicle-treated ultrasound-exposed group, one-way ANOVA and post hoc Tukey’s test). 6-11 animals per group were used. Doses are expressed in mg/kg per day. Bars are mean ± SEM.

| A 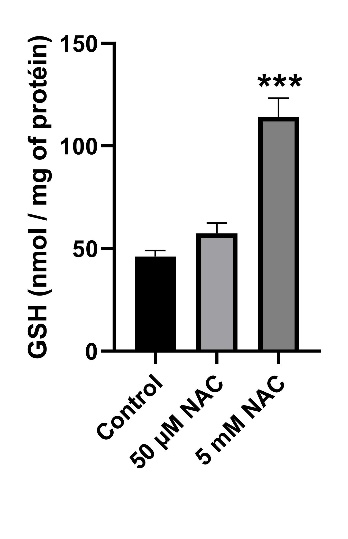 | 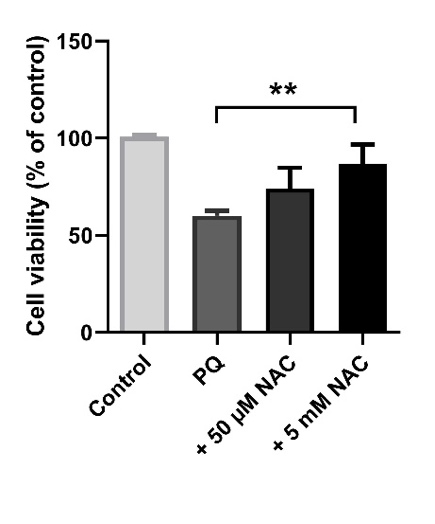B | 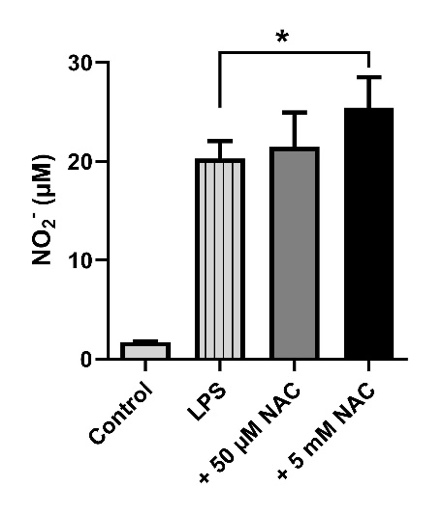C |
| --- | --- | --- |

**Supplemental figure 16: Effect of N-acetylcysteine (NAC) on the levels of GSH in Neuro2a cells (A) and cell viability of PQ-challenged Neuro2a (B) and the production of NO_2_^-^ in LPS-challenged BV2 cells (C).** The cells were incubated for 25 h in the presence of 5 µM or 5 mM N-acetylcysteine. PQ (0.25 mM, 24 h) or LPS (1 µg/ml, 24 h) were added after 1 h The data are expressed as mean ± SD (n=3). (n=3, *, p<0.05, **, p<0.01, ***, p<0.001, ANOVA followed by a Tukey post-hoc test.
